# Revisiting the Importance of Orthobunyaviruses for Animal Health: A Scoping Review of Livestock Disease, Diagnostic Tests, and Surveillance Strategies for the Simbu Serogroup

**DOI:** 10.3390/v16020294

**Published:** 2024-02-15

**Authors:** Tiffany W. O’Connor, Paul M. Hick, Deborah S. Finlaison, Peter D. Kirkland, Jenny-Ann L.M.L. Toribio

**Affiliations:** 1 Sydney School of Veterinary Science, Faculty of Science, The University of Sydney, Camden, NSW 2570, Australia; jenny-ann.toribio@sydney.edu.au; 2 Virology Laboratory, Elizabeth Macarthur Agricultural Institute, NSW Department of Primary Industries, Menangle, NSW 2568, Australia; paul.hick@dpi.nsw.gov.au (P.M.H.); deborah.finlaison@dpi.nsw.gov.au (D.S.F.); peter.kirkland@dpi.nsw.gov.au (P.D.K.)

**Keywords:** arboviruses, animal health, orthobunyavirus, Simbu serogroup

## Abstract

Orthobunyaviruses (order *Bunyavirales*, family *Peribunyaviridae*) in the Simbu serogroup have been responsible for widespread epidemics of congenital disease in ruminants. Australia has a national program to monitor arboviruses of veterinary importance. While monitoring for Akabane virus, a novel orthobunyavirus was detected. To inform the priority that should be given to this detection, a scoping review was undertaken to (1) characterise the associated disease presentations and establish which of the Simbu group viruses are of veterinary importance; (2) examine the diagnostic assays that have undergone development and validation for this group of viruses; and (3) describe the methods used to monitor the distribution of these viruses. Two search strategies identified 224 peer-reviewed publications for 33 viruses in the serogroup. Viruses in this group may cause severe animal health impacts, but only those phylogenetically arranged in clade B are associated with animal disease. Six viruses (Akabane, Schmallenberg, Aino, Shuni, Peaton, and Shamonda) were associated with congenital malformations, neurological signs, and reproductive disease. Diagnostic test interpretation is complicated by cross-reactivity, the timing of foetal immunocompetence, and sample type. Serological testing in surveys remains a mainstay of the methods used to monitor the distribution of SGVs. Given significant differences in survey designs, only broad mean seroprevalence estimates could be provided. Further research is required to determine the disease risk posed by novel orthobunyaviruses and how they could challenge current diagnostic and surveillance capabilities.

## 1. Introduction

Arboviruses within the *Orthobunyavirus* genus (order *Bunyavirales*, family *Peribunyaviridae*) pose a substantial animal health threat as the geographical spread of insect-borne viruses expands and the frequency and magnitude of their epidemics increase [1,2]. Orthobunyaviruses are approximately 90–100 nm in diameter, spherical, and double-membraned with a fringe of protein spikes [2]. The glycoprotein spikes are arranged in an ordered lattice and protrude 18 nm from the surface [2]. The genome consists of three separate, single-stranded, negative-sense RNA segments that are named according to their size: small (S, ~1 kb), medium (M, ~4.5 kb), and large (L, ~6 kb) [3]. Several orthobunyaviruses, including Schmallenberg virus (*Orthobunyavirus schmallenbergense*) and Akabane virus (*Orthobunyavirus akabaneense*), have been responsible for widespread epidemics of congenital disease in ruminants [2].

Taxonomic classification of orthobunyaviruses is challenging because of the genetic diversity and the large number of viruses in the genus. Historically, many orthobunyaviruses were isolated through international discovery programs and named based on their antigenic similarity [4,5]. Their antigenic properties are related to the two surface glycoproteins (Gn and Gc) and the nucleoprotein (N) protein encoded by the M and S segments, respectively [6]. The genus was organised into 18 serogroups based on antigenic similarities between viruses [5,7]. The current taxonomic classification system is based on the evolutionary history of the viruses [8,9]. Orthobunyaviruses are grouped into species complexes when there is ≥96% identity in the complete amino acid sequence of the L protein, an RNA-dependent RNA polymerase translated from the L segment [8,10]. As a result, species complexes have grouped together multiple viruses with different antigenic characteristics [11] (Supplementary Table S1). This genus currently comprises over 189 named viruses assigned into 103 species complexes [12].

The Simbu group is one of the larger serogroups, comprising 33 named viruses assigned to 19 species complexes with a global distribution (Supplementary Table S1) [5,8]. The Simbu group viruses (SGVs) may be further genetically divided into two monophyletic clades, Clade A and Clade B. Clade designation is based on phylogenetic relationships between SGVs and reflects an evolutionary divide that is consistent with previously established serogroups [13]. Ladner et al. 2014 present a phylogenetic tree depicting the separation between clades. Serogroup and clade assignment allows for virus classification by both their antigenic and phylogenetic properties [11,13]. The vertebrate hosts, vectors, and/or pathogenicity of over 60% of the SGVs within Clade A remain unknown (Supplementary Table S1). Several Clade A viruses are classified in the Oropouche species complex (*Orthobunyavirus oropoucheense*), and notably, two are associated with fever and encephalitis in humans. Clade B comprises viruses that are important animal pathogens, including Akabane virus (*Orthobunyavirus akabaneense*), Schmallenberg virus (*Orthobunyavirus schmallenbergense*), and Shuni virus (*Orthobunyavirus shuniense*) [14,15,16].

**Figure 1:**
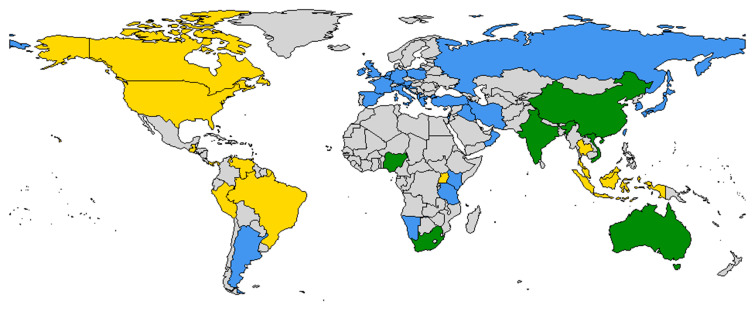
The distribution of the orthobunyaviruses within the Simbu group; Clade A viruses were reported within the countries in yellow, Clade B viruses in countries in blue, and viruses from both clades were reported within countries in green.

For SGVs in Clade B, virus transmission to mammalian hosts occurs via hematophagous arthropods such as biting midges of the genus *Culicoides* (Supplementary Table S1). These Clade B viruses, several of which are recognised teratogens, have been found worldwide, with the exception of North America (Figure 1, Supplementary Table S1). In endemic regions, virus infection typically results in minimal disease in adult animals during transmission seasons of high vector activity. However, epidemics occur when the distribution of the insect vector intermittently overlaps with that of a naïve host population [17,18]. This occurs when environmental conditions allow the insect vectors to move into new populations of susceptible livestock or when these insect vectors are absent from part of their normal distribution range for some time, giving rise to susceptible mammalian hosts [18]. The most severe disease syndrome is observed when naïve pregnant animals are infected at critical stages of gestation resulting in foetal pathology following in utero infection.

In Australia, the distribution of selected insect-borne viruses of veterinary importance is monitored by the National Arbovirus Monitoring Program. This active surveillance system involves the longitudinal sampling of sentinel herds across Australia and trapping *Culicoides* midges [19]. Data on both population immunity and vector activity can be used to identify areas free from arbovirus transmission, where naïve animals may be at risk of disease, and to support trade. In January 2017, an unknown orthobunyavirus was detected in a sentinel cattle herd during surveillance activities in New South Wales (NSW), Australia. Preliminary analyses suggest that this isolate may be most similar to Shamonda virus (*Orthobunyavirus schmallenbergense*). To inform the priority that should be given to the detection of a Shamonda-like virus in Australia, a scoping review was undertaken to (1) characterise the associated disease presentations and establish which of the Simbu group viruses (SGVs) are of veterinary importance; (2) examine the diagnostic assays that have undergone development and validation for this group of viruses; and (3) describe the methods used to monitor the distribution of the SGVs.

## 2. Materials and Methods

### 2.1. Data Collection

The review protocol was completed in accordance with the Preferred Reporting Items for Systematic Reviews and Meta-Analyses extension for Scoping Reviews guidelines [20]. The protocol was agreed upon by all authors on 28 April 2022.

Several combinations of search terms were attempted to identify eligible publications with no restrictions applied to search fields. Initially, given the detection of a Shamonda-like virus, a search to identify publications describing SGVs and, specifically, for this virus was used to frame the study objectives. This first strategy used the genus name but restricted publications to those including the serogroup name or the virus name. However, to encompass the whole Simbu group, this strategy was expanded to include varying combinations of genus and individual virus names. This second search strategy listing the genus name and all 33 virus names returned 276,211 publications in Scopus. This search strategy was refined further by restricting to domestic animal species of interest only (bovine, ovine, caprine, equine, and swine). This was done to limit this scoping review to a manageable size based on economically important domestic agricultural species. This refined search strategy returned 8677 publications in Scopus before it was used across other scientific databases. Both search strategies were combined to identify publications in five databases: Scopus, Web of Science, CAB Abstracts, BIOSIS and Medline. The first search was performed on 26 May 2022. The second search was performed on 11 August 2022. The search was applied across all databases, and the publications retrieved from both strategies were exported into the citation manager software Endnote(X9).

**Figure 2:**
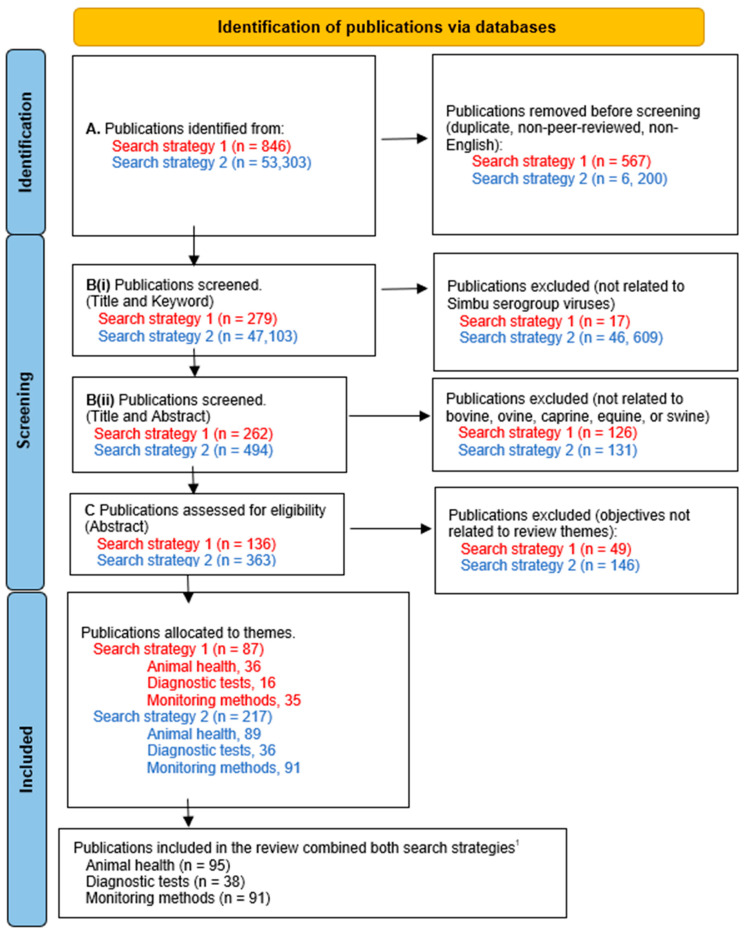
Workflow used to screen for eligible literature in this scoping review to determine the animal disease, diagnostic tests, and monitoring methods for orthobunyaviruses in the Simbu group with veterinary importance. ^1^ Eight publications were incorporated from the first search strategy, which were not identified via the second approach.

A systematic approach was applied to find eligible publications using three levels of screening (Figure 2):

(A) Screening by publication type for original research publications in English, with no restrictions on publication date and type. Duplicates and non-peer-reviewed publications were excluded.
(B) Screening by title and keywords for eligible publications relevant to the 33 viruses of the Simbu group and by title and abstract for domestic ruminant, equid, or porcine species.
(C) Screening by abstract for eligible publications providing information relevant to at least one of the three themes of interest. Allocation to a theme was based on the aims and objectives of the research publication. Where the publication could be relevant to more than one theme, allocation was made following evaluation of the text in full.

Data were extracted for all three themes according to the details listed in Supplementary Table S2:

(1) Animal disease presentation, which recorded observations of clinical signs associated with SGVs. An assessment was completed to ensure that cases of clinical disease described were confirmed to be a result of infection with the virus of interest. The diagnostic criteria are based on the unified case definitions proposed by European agencies during data collection for the Schmallenberg virus epidemic [21]. The criteria for case definition were as follows:
  (a) Clinical presentation only;
  (b) Antibody detection only and/or agent detection only;
  (c) Consistent clinical presentation with confirmation of viral infection with an SGV.
(2) Diagnostic tests, which described the development or validation of tests for detecting SGVs.
(3) Monitoring, which determined the distribution of SGVs. A quantitative summary of seroprevalence by country, animal species, and Simbu group virus was prepared using R with data extracted.

### 2.2. Data Analysis

The data were recorded in Excel (Microsoft, 2023) before data summaries were prepared in R (R Core Team, 2022). For publications where multiple categorical levels of one variable were applicable (e.g., host species had >1 species listed if an eligible publication described disease presentation in multiple species), the details were recorded as a comma-separated list. Vector operations, grouped into the ‘RepRows’ function, in R were used to extract the recorded individual items in a free text list and to match these variables to the details of each publication. A record (‘n’) was generated for each extracted item in the free text list from each publication. This allowed for multiple recorded items for the same variable to be counted and analysed from the one publication (i.e., for multiple host species, a separate record for bovine and ovine would be generated for the same publication). To avoid double counting, multiple variables were not extracted together for analysis.

## 3. Results

### 3.1. Selection of Sources of Evidence

The implementation of the protocol and screening processes identified 87 eligible publications from the first search strategy and 217 publications from the second search strategy (Figure 2). Eight publications were incorporated through the first strategy that were not identified via the second approach. Most publications (n = 46,609) were removed in the second search strategy after title and keyword screening for relevance (Figure 2). A total of 224 publications were combined from both search strategies, with 95 publications allocated to the animal disease presentation, 38 publications for diagnostic test development and validation, and 91 publications for monitoring methods to determine SGV distribution.

### 3.2. Characteristics of Sources of Evidence

The 224 eligible peer-reviewed publications were retrieved from 70 different journals. The five journals with the most publications were: *Transboundary and Emerging Diseases* (n = 23), *Veterinary Microbiology* (n = 19), *Emerging Infectious Diseases* (n = 12), *Australian Veterinary Journal* (n = 11), and *BMC Veterinary Research* (n = 10). The authors of the publications represented 50 countries, with most from Japan (n = 45), Germany (n = 30), Australia (n = 28), Belgium (n = 23), and France (n = 18).

**Figure 3:**
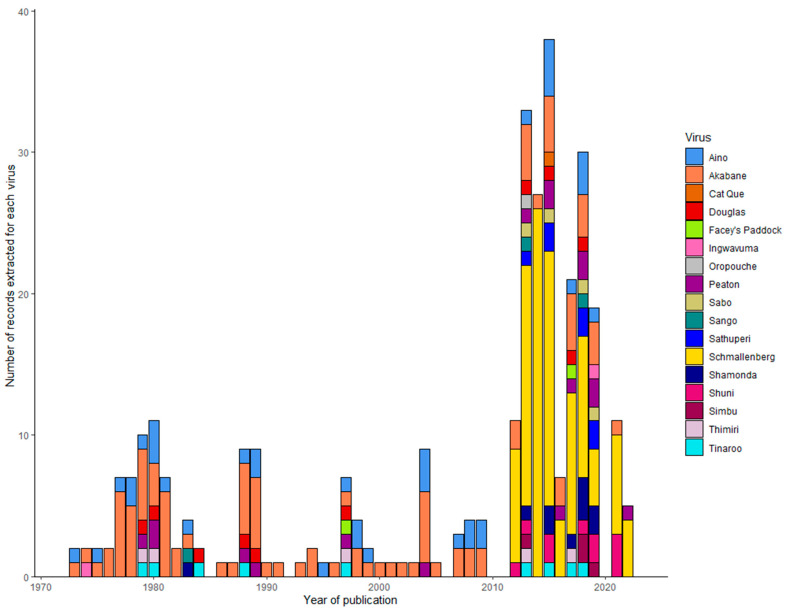
The publication year and number of records for viruses in the Simbu group in the 321 records extracted from the 224 eligible publications retrieved from the scoping review protocol.

A total of 321 records were extracted by virus name from all 224 eligible publications. Overall, Schmallenberg virus was the most frequently investigated (n = 108), followed by Akabane virus (n = 95), Aino virus (n = 37), and Peaton virus (n = 17). Following these, four viruses, Shuni virus, Shamonda virus, and Douglas virus, were investigated with equal frequency (n = 10). Records encompassed 17 of the 33 named viruses within the Simbu group. This included 10 viruses investigated for animal health impacts, 13 viruses for monitoring distribution, and 16 viruses for diagnostic tests (Table 1). The publications spanned from 1973 to 2022. Records for both Akabane virus and Aino virus were consistently published over this 49-year period (Figure 3).

### 3.3. Animal Health Impacts Associated with Simbu Group Viruses

#### 3.3.1. Clinical Presentation of Livestock Disease

Overall, congenital malformations in ruminants were the most frequently observed disease syndrome attributed to SGV infection. Disease observations were most frequently recorded for bovines and most often with Akabane virus. Six SGVs resulted in congenital malformations in cattle: Akabane virus, Schmallenberg virus, Aino virus, Shuni virus, Peaton virus, and Shamonda virus. In contrast, only three SGVs (Akabane virus, Schmallenberg virus, and Shuni virus) were associated with congenital malformations in sheep and goats. The type of congenital malformations depended on the gestational age at the time of infection (Table 2).

Most reports of severe neurological disease involved calves following in utero infection with Akabane virus at one of two distinct periods of gestation (Table 3). Neurological signs were also reported in calves born alive following in utero infection with Shuni virus [34,35], Aino virus [36,37], Peaton virus [38], or Schmallenberg virus [39]. Neurological signs were also reported in lambs following in utero infection with Akabane virus [40,41]. The neurological signs included dysstasia/astasia, ataxia, circling, dull/slow to respond to surroundings, proprioceptive deficits, nystagmus, hypersensitivity, paralysis, tremors, abnormal gait, recumbency, unconscious paddling, loss of proprioception, swallowing difficulties, and tongue paralysis. The musculoskeletal signs (stiff joints, lameness) were only reported in conjunction with neurological disease in calves and lambs following in utero infection from Schmallenberg virus or Akabane virus [39,42,43].

Reproductive disease in adult animals was also observed across ruminant species and included reports of early embryonic death, repeated oestrus, and increased return to inseminations. Again, most disease was reported for cattle, with reproductive diseases also associated with five SGVs: Schmallenberg virus, Akabane virus, Aino virus, Shuni virus, and Peaton virus. For sheep and goats, the same three SGVs (Schmallenberg virus, Akabane virus, and Shuni virus) were reported in association with both congenital malformations and reproductive signs. In comparison with congenital malformations, reproductive disease observations were most often associated with Schmallenberg virus across all three ruminant species. This strong association between Schmallenberg virus and reproductive disease is most obvious in sheep.

Non-reproductive disease in horses and adult ruminants was uncommon. There were five publications describing disease in adult animals attributed to infection with an SGV. Although neurological disease in horses following Shuni virus infection is the most common disease presentation in adult animals [16,44], there were three case reports of neurological disease in adult cattle following infection with Shuni virus [34] and Akabane virus [45,46]. The neurological signs were comparable between juvenile and adult animals (Table 3).

**Figure 4:**
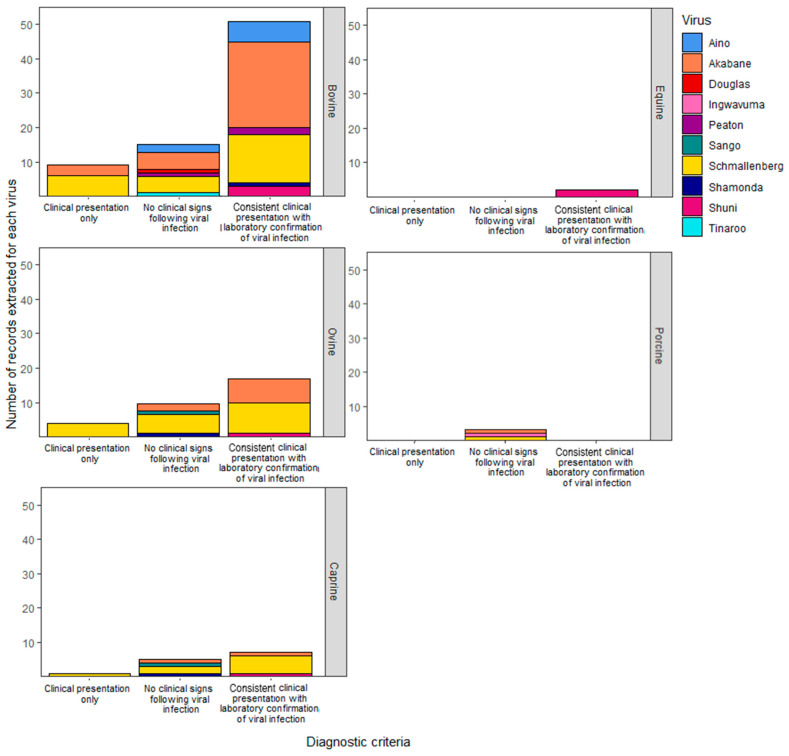
Simbu group viruses investigated in disease reports in different livestock species. The four diagnostic criteria were based on case definitions provided by European agencies during data collection for the Schmallenberg virus epidemic [21] with antibody-detection-only and agent-detection-only categories combined as ‘No clinical signs following viral infection’ for this figure.

Other disease observations in horses and adult ruminants included non-specific signs, including pyrexia, reduced appetite with subsequent weight loss, temporarily reduced milk yield, and excess salivation. Diarrhoea, the only gastrointestinal clinical sign observed, was reported in adult cattle and sheep following natural and experimental infection with Schmallenberg virus [47,48,49]. Nasal discharge and tachypnoea were the only two respiratory signs observed. These subtle signs were observed only during experimental infection with Schmallenberg virus in sheep [49] or Akabane virus in cattle [50]. There were no cases of clinical disease for swine examined in this review. This is because the two records retrieved following infection with Cát Quế virus and Ingwavuma virus in this systematic search did not fulfil the case definition required (Figure 4).

Disease was reported more often in winter (n = 26), spring (n = 22) and autumn (n = 20) than in summer (n = 12). This seasonal trend was consistent across all countries and viruses investigated, apart from Shuni virus. For Shuni virus, disease was more often reported in summer (n = 3) and autumn (n = 3) than in winter (n = 1) and spring (n = 1). The case numbers and frequency of clinical signs were extracted from 37 publications to provide the descriptive summary below (Table 3). A histopathological description was provided in some publications describing diseases in ruminants (29/95, Supplementary Table S3).

In addition to the loss of replacement animals, economic impacts associated with disease from SGVs are due to costs associated with veterinary treatment and reduced production. Replacement animal costs represent a substantial economic loss. The Schmallenberg virus outbreak in Ireland in 2012 resulted in an estimated 10% decrease in weaning rates for affected sheep farms [51]. Veterinary costs were attributed to dystocia, which is common for ruminants following abortion, stillbirth, or the attempted birth of a malformed foetus. Direct veterinary costs were estimated to be EUR 65–107 per animal affected in Belgium in 2011 [52]. In dairy cows experiencing dystocia, milk yield decreased by 11.4–24.7% compared with normal parturition [53]. Adding to these production losses, acute infection in adult ruminants may result in reduced milk yield (Table 3). Overall, the economic impact of disease associated with Schmallenberg virus in cattle and sheep was best summarised in two European studies. In 2015, estimated disease costs per cow per year were GBP 9.7–48.6 in France and GBP 8.2–51.4 in the UK for cattle [54]. In 2017, the estimated disease cost per cow per year was EUR 23–43 in France [55]. This 2017 study also estimated the disease costs per ewe per year to be EUR 19–37 in France [55].

#### 3.3.2. Simbu Group Viruses Associated with Livestock Disease

Most of the publications were observational studies (66/95) encompassing descriptive case series (n = 25), cross-sectional studies (n = 16), single case reports (n = 15), case–control studies (n = 4), and cohort studies (n = 2). Just over two-thirds of the publications fulfilled at least one of the four diagnostic criteria for Simbu group virus infection (61/95, Figure 4). Akabane virus was extensively studied as a causative agent of animal disease. There are 31 records where clinical disease caused by Akabane virus infection was diagnosed. Five other Simbu group viruses met the required case definition, including Schmallenberg virus (n = 19) and then Aino virus (n = 6), Shuni virus (n = 5), Peaton virus (n = 2), and Shamonda virus (n = 1). While Ingwavuma virus, Douglas virus, Tinaroo virus, and Sango virus were also investigated, these viruses were not diagnosed as the cause of clinical disease in livestock (Figure 4).

Disease was observed following experimental infection for four SGVs from 29 publications: Akabane virus (n = 16), Schmallenberg virus (n = 10), Aino virus (n = 2), and Shuni virus (n = 1). Inoculation was most often by subcutaneous (n = 11) or intravenous (n = 11) injection in pregnant animals, resulting in congenital malformations of the developing foetus. Other successful routes include intramuscular injection (n = 2), oronasal administration (n = 1), and intrauterine inoculation (n = 1), including intra-peritoneal injection of the foetus (n = 1). Intracerebral injection (n = 2) of juvenile calves resulted in the development of neurological disease with Akabane virus [43] and non-specific signs such as lethargy and pyrexia with Aino virus [36]. No clinical disease was reported following the experimental challenge (n = 21) of non-pregnant adult ruminants with Shamonda virus, Douglas virus, Sango virus, or Peaton virus despite laboratory confirmation of viral infection by agent or antibody detection (Figure 4).

Laboratory tests for infections with SGVs in the 95 publications describing livestock disease presentation involved a range of different assay types. The earliest diagnostic tests used were virus neutralisation tests (VNTs), virus isolation, immunofluorescence testing (IFAT), and histopathological examination. The earliest ELISA tests for SGVs were used in a publication from 1988 [41], and PCR diagnostics tests became available from 2002 [46]. Supportive evidence for viral infection was provided through a range of assays: PCR (n = 11), VNTs (n = 11), ELISAs (n = 4), histopathological examination (n = 4), or virus isolation (n = 2). In over half the publications (n = 50), a combination of assay types was utilised to provide confirmation of viral infection. This included a combination of the aforementioned assays and/or the following: IFAT; immunohistochemistry; the agar gel immunodiffusion test (AGID); the complement fixation test (CFT); in situ hybridisation; nucleic acid sequencing; and foetal IgG, IgA, or IgM testing. The most frequent combinations included two test types—VNTs with virus isolation (n = 7), ELISAs with PCR (n = 5), and VNTs with histopathological examination (n = 4). Thirteen publications described the clinical presentation only and did not provide the details of laboratory testing (Figure 4).

### 3.4. Test Development and Validation Studies for Assays to Detect Viruses in the Simbu Group

Out of the 38 publications for review, 23 described assay development, and the remaining 15 included descriptions of test validation. Validation, according to the World Organisation for Animal Health (WOAH) guidelines, was completed to Stage 1 for 8/15 publications, where the study determined analytical sensitivity and specificity and demonstrated repeatability and reproducibility compared with standard test methods [56]. Validation was completed to Stage 2 for 7/15 publications, where the study established the diagnostic sensitivity and specificity of the candidate test on a reference population and determined cut-off values [56].

Most publications described an assay designed for the purpose of diagnosis (33/38) where a disease was suspected to be a consequence of SGV infection. Diagnostic tests were most often developed for Schmallenberg virus (n = 21) and Akabane virus (n = 20) (Table 1). Assays in development were more often designed to detect a specific named virus (31/38) rather than a pan-reactive group test (7/38). Only a few described an assay that was used for surveillance purposes (5/38), where testing is completed in situations where there is no suspicion of disease. Given this limited validation of diagnostic tests for surveillance purposes, most of the assays were designed for use with individual animal samples (36/38). Only 2 out of 38 assays were designed for pooled samples for herd or population testing. In these instances, bulk milk was used as a population sample for developing serological tests.

Blood, whether in the form of serum (n = 22), whole blood (n = 9), or substitutes like thoracic fluid (n = 1), was the most used sample type for developing and validating assays. Although blood is an obvious choice for serological testing, publications were almost evenly split between tests targeting antibody (18/38) or agent (17/38) detection. The agent detection assays also used a range of tissues as sample types in test development and validation (n = 14), with the brain being the most frequently used organ (n = 5). Semen (n = 2), *Culicoides* homogenates (n = 2), and milk (n =1) were less often utilised.

The assay types that were most often evaluated were PCR (n = 18), ELISAs (n = 18), and VNTs (n = 17). Less commonly evaluated assay types included the haemagglutination inhibition test (n = 2), electron microscopy (n = 1), and IFAT (n = 1). Novel technologies were also investigated, such as loop-mediated isothermal amplification (LAMP) (n = 2), recombinase polymerase amplification (n = 1), and antibody detection with the Luminex MAGPIX^®^ system (n = 1). Further details on the test components were available for 30/38 publications, allowing for the details on test design to be presented below.

#### 3.4.1. Agent Detection

Molecular assays for agent detection were most often designed with amplification targets in the S segment (n = 17) compared with the M (n = 2) and L segments (n = 3). The full details on the primers and probe sequences are provided in Supplementary Table S4. Specific PCR assays for named viruses within the serogroup were mostly developed for Akabane virus (n = 8), Schmallenberg virus (n = 5), and Aino virus (n = 5). The S segment was the target of most of these specific virus assays (n = 15). The M segment is a highly variable genomic region, and this segment was used in the development of a virus-specific PCR for Peaton virus [57] and Schmallenberg virus [58]. Two virus-species-specific PCRs based on the L segment were described for Schmallenberg virus [58,59,60]. To develop a group-reactive PCR, primer and probe sequences were designed for the S [57,61,62] and L segments [58,63,64].

To achieve WOAH Stage 1 validation, known virus isolates were utilised in PCR development to demonstrate analytical specificity against the different SGVs and analytical sensitivity with dilution series conducted to determine the limit of detection (Table 1). Only two PCR assays were validated with reference samples to establish diagnostic specificity and sensitivity [62,65]. Reference samples in both publications were limited to materials with Schmallenberg virus with known positive samples sourced from experimentally infected animals and/or field samples. However, the diagnostic specificity could not be estimated for the Schmallenberg virus real-time PCR assay (SBV-S) because no known negative samples were tested in this publication. During testing to determine the analytical specificity of the SBV-S assay, false-positive results were observed for five out of nine virus isolates, which included Sabo virus, Shamonda virus, Simbu virus, Peaton virus, and Douglas virus. Nonetheless, the SBV-S PCR test has a diagnostic sensitivity of 100% when assessed using reference samples that are positive for the Schmallenberg virus (91.8–100%, n = 43) [65]. The universal S-segment-based real-time PCR assay (Uni-S) has a diagnostic specificity of 100% (89.1–100%, n = 32) and a diagnostic sensitivity of 83.7% (69.3–93.2%, n = 43) [62] when assessed using reference samples for Schmallenberg virus, Akabane virus, and Shuni virus.

#### 3.4.2. Antibody Detection

For ELISAs, the detection of orthobunyaviruses within the same serogroup is achieved when the test is designed against the N protein. However, the detection of a specific virus within the serogroup can be achieved when the Gc protein is used as the test target, such as for Akabane virus [66]. Details were available across 11 publications, extracted into 15 records, for ELISAs designed to detect antibodies for Schmallenberg virus or Akabane virus. Six commercially available ELISAs were described (Table 4). General designs for commercial antibody tests included competition and indirect ELISA formats utilising N protein (n = 3), Gc protein (n = 1), or whole virus antigens (n = 2) to detect antibodies. An in-house ELISA was described in four publications (n = 1 for each) [67,68,69,70].

To achieve WOAH Stage 1 validation, hyperimmune sera against known viruses were utilised in the ELISA development to demonstrate analytical specificity. For Stage 2 validation, most publications utilised reference samples taken from field submissions that were tested using a VNT for Schmallenberg virus, Akabane virus, or Shuni virus (Table 1). In addition to validation testing completed for the commercial assays (Table 4), an ELISA test to detect specific viruses in the Simbu serogroup showed overall diagnostic specificities of 84.56%, 94.68%, and 89.39% and sensitivities of 89.08%, 69.44%, and 84.91% for Schmallenberg virus, Akabane virus, and Shuni virus, respectively [66]. The test was conducted on a sample of 477 known negative cases and 238 known Schmallenberg-virus-antibody-positive, 36 known Akabane-virus-antibody-positive, and 53 known Schmallenberg-virus-antibody-positive cases.

### 3.5. Monitoring the Distribution of Viruses in the Simbu Group

Across the 91 publications describing the distribution of SGVs, Schmallenberg virus (n = 51) was the most studied, followed by Akabane virus (n = 32) and Aino virus (n = 21) (Table 1). Most studies were conducted within a country (n = 59) or local region (n = 24) rather than across multiple countries (n = 6) or continents (n = 2). Overall, the monitoring methods described were conducted across 56 countries and covered all continents except North America. The countries most frequently studied were Australia (n = 9), Korea (n = 9), Belgium (n = 8), Japan (n = 8), Germany (n = 5), the Netherlands (n = 5), Türkiye (n = 5), China (n = 4), France (n = 4), and Ireland (n = 4).

Most publications described active sample collection (n = 70) rather than the opportunistic use of existing samples (n = 20) collected in surveillance for other viral pathogens. One publication described a mixed approach to SGV surveillance. Most surveys (n = 66) aimed to detect only one virus. Some investigated a combination of SGVs including other animal pathogens (n = 20). In some cases, the publication described a general arbovirus survey where an SGV was detected without specifying the virus of interest (n = 5).

In over half the publications, samples were collected from a single mammalian host species, bovine (n = 35), ovine (n = 5), caprine (n = 3), equine (n = 2), and porcine (n = 1), or samples of vectors, such as *Culicoides* spp. (n = 2). In 43 publications, samples were collected from a combination of animals and insect species. This included a combination of two or more ruminant species (n = 27) or a combination of a ruminant and other host species such as camelids; swine or equines (n = 6); or finally, a combination of a mammalian species and *Culicoides* (n = 10).

Overall, a single sample type was also most often collected: serum (n = 67), milk (n = 6), or *Culicoides* (n = 2). Occasionally, a combination of these sample types (n = 8), including aborted foetal tissues (n = 4), EDTA-treated blood (n = 3), and vaginal swabs (n = 1), were utilised. Milk yield data from dairy cattle was used in a novel approach to provide an early warning system for Schmallenberg virus transmission [76].

Both agent and antibody assays were utilised as part of the efforts to detect SGVs and determine the distribution of these viruses. Most publications described the use of only one type of assay in their testing method. In these circumstances, antibody tests such as ELISAs (n = 32) and VNTs (n = 23) were more often used rather than agent-specific PCRs (n = 2). Less than a third of publications (n = 24) utilised a combination of two assay types to both detect and distinguish the SGV surveyed, including the following:

(A) Multiple antibody tests—ELISAs with VNTs (7/24) or haemagglutination inhibition tests (HITs) with VNTs (3/24);
(B) A combination of both antibody and agent test types—ELISAs with PCR (9/24) or VNTs with PCR (2/24);
(C) A specific antibody test and general assay such as a VNT with virus isolation (3/24).

More than three tests were combined with varying permutations of assay types in 10 publications. These combinations utilised an agent-specific test, such as PCR (7/10), with antibody assays, including ELISAs (6/10), VNTs (9/10), HITs (1/10), IFAT (2/10), the AGID (2/10), and the CFT (1/10), and general test types such as virus isolation (4/10) and electron microscopy (1/10).

Only one publication conducted a survey with the purpose of demonstrating proof of freedom. Instead, almost all publications were conducted with the intent of virus detection. As such, most samples were collected at a single time point (62) rather than repeated (27). For repeated sampling, a monthly period was the most frequent interval (16), followed by quarterly (5) and yearly (2). Daily (1) and weekly (1) sampling was uncommon. Sampling was also repeated following specific circumstances such as calving (2). The information on sampling interval was not available for two publications. Autumn was most often the season in which sampling commenced (15/48), closely followed by spring (13/48), summer (12/48), and then winter (8/48). The median sampling period was 14.38 months (minimum, 1 month; mean, 6 months; maximum, 108 months) described in 32/91 publications.

Overall, the median total number of animals sampled for over a third of all publications (71/91) was 507 (min., 9; Q1, 239; mean, 2285; Q3, 1936; max., 21,833). Sample size calculations were reported in almost one-third of the publications (25/91). Where sample size calculations were made, the median sample size was 1301 (min., 42; Q1, 361; mean, 3647; Q3, 4670; max., 21,833). The assumptions in survey design were most varied based on the anticipated prevalence (median, 20%; range, 1–98%), whereas error (median, 5%; range, 1–15%) and confidence (median, 95%; range, 95–99%) were within a narrow range. Based on the seroprevalence measure reported across 57 publications, overall, the median seroprevalence was 18.8% (min., 0; Q1, 2.9%; mean, 28.9%; Q3, 48%; max., 100%). The mean seroprevalence was highly varied and is presented by country, animal species, and virus in Supplementary Figure S1.

## 4. Discussion

Orthobunyaviruses in the Simbu group can pose a significant threat to livestock production. The emergence of these viruses, in particular Akabane virus and Schmallenberg virus, in naïve populations has led to epidemics with profound animal health and welfare impacts. This scoping review covers almost five decades of research on SGVs and presents the anticipated disease presentations of veterinary-important orthobunyaviruses in livestock, with considerations regarding the published diagnostic assays and surveillance strategies used to monitor the distribution of Simbu group viruses. The findings justify the need to revisit these viruses as re-emerging pathogens with significant animal health impacts.

This review examined the literature from 1973 to 2022. The peak output years of publications were associated with the occurrence of epidemics caused by SGVs (Figure 3). This includes publications from the 1970s to 1980s, associated with epidemics of Akabane virus in Australia, Japan, and Israel, and from 2011 in the decade following the emergence of Schmallenberg virus across Europe [15]. The disease epidemics associated with these SGVs are not the only explanation for the expansion of publications from 2011 (Figure 3). More viruses in this serogroup are being recognised as causes of disease in livestock. For example, Shuni virus was first isolated from a healthy cow in Nigeria in 1966 [77]. Reports of neurological disease in horses associated with Shuni virus were much later investigated in South Africa in 2009 [16]. Shuni virus has been linked to congenital malformations and reproductive disease in cattle, sheep, and goats in Israel between 2014 and 2015 [35] and also caused neurological disease in a 16-month-old heifer in Israel in 2016 [34]. Peaton virus was first isolated from a healthy cow in Australia in 1979 [78] and was recently recognised as a cause of congenital malformations in calves in both Japan and Israel in 2018–2019 [38,79]. Finally, Shamonda virus was first isolated from cattle in Nigerian markets in 1965 [77]. Serological evidence suggests that Shamonda virus was a cause of congenital malformations in calves in Japan in the early 2000s [80,81]. More recently, Shamonda virus was detected in cases of febrile illness associated with abortions in goats in South Africa in 2023 [82]. The detection of a Shamonda-like isolate during surveillance activities in NSW provides the justification to revisit SGVs and investigate their potential impacts on livestock health in Australia.

Congenital malformations in ruminants, particularly in cattle, were identified as the most frequent disease manifestation of viruses within the Simbu group. Gestational age determines the form of congenital malformations observed (Table 3). The pathogenesis of congenital disease was determined during outbreak investigations of Akabane virus in Australia and Japan [83,84] and has been well characterised by experimental infection studies [22,23,28,29,31]. This observation was further confirmed during the Schmallenberg virus epidemic that occurred across Europe [85]. Infection before embryonic attachment may also cause embryo loss, resulting in observations of reproductive disease in adult animals [86,87]. Following foetal infection, neuroglial cells appear to be the sites of virus predilection, as do neuronal cells in the spinal ventral horn [88]. If the developing foetus survives, this results in pathological changes in the brain and spinal cord. If infected mid-gestation, lesions in the central nervous system also result in musculoskeletal changes due to a lack of movement in the developing foetus. The developing skeletal muscle may also be infected and is often atrophied and replaced by adipose tissue [22]. In the late gestation and perinatal periods, following the development of immunocompetence, infection may lead to inflammatory changes in the central nervous system. If the neonate is not aborted, survives to term, and is not stillborn, encephalomyelitis may be observed with neurological signs.

While reviewing the frequency of clinical signs and necropsy findings for each virus within the Simbu group, it became evident that orthobunyaviruses in this serogroup exhibit varying propensities for foetal pathology (Table 3). Akabane virus is more often associated with congenital malformations, while Schmallenberg virus is found to frequently affect reproductive outcomes. In previous experimental infection challenges that utilised different strains of the same named virus within this serogroup, it was shown that there could be varying propensities for disease expression. The differences in the pathogenicity of strains are most often reported for Akabane virus isolates. A mouse model experiment demonstrated the differences in the neuroinvasiveness and neurovirulence of Akabane virus isolates [89]. These strain differences were also observed in a comparison of teratogenicity observed in ovine foetuses following separate experimental inoculations conducted in Japan and Australia [28,31]. The degree of foetal pathology observed was influenced by the number of passages in cell cultures and the cell lines used [38], and this method has been utilized in the production of a vaccine for Akabane virus [90,91]. The determinants of pathogenicity for SGVs have not been identified, although some elements of pathogenesis have been characterised, such as the ability to induce viraemia in a mammalian host [92,93]. Further investigation into the molecular relationships between pathogenic and non-pathogenic viruses could help define the genetic determinants of disease. Knowledge of the drivers for pathogenicity would be especially useful for selecting safe vaccines and also allows for virulence to be monitored during production [94].

Out of the 33 named viruses in this serogroup, only 6 were identified as a cause of disease in livestock: Akabane virus (*Orthobunyavirus akabaneense*); Schmallenberg virus and Shamonda virus (*Orthobunyavirus schmallenbergense*); Aino virus (*Orthobunyavirus ainoense*); Shuni virus (*Orthobunyavirus shuniense*); and Peaton virus (*Orthobunyavirus peachesterense*). All are phylogenetically classified into Clade B. However, there are other named virus entities within this phylogenetic structure that are closely related but have no recognised disease manifestations in livestock (Table 3). This includes Tinaroo virus and Yaba-7 virus (both belonging to the species *Orthobunyavirus akabaneense*); Sathuperi virus and Douglas virus (both *Orthobunyavirus schmallenbergense*); and Kaikalur virus (*Orthobunyavirus shuniense*). To gain a better understanding of the risks posed by orthobunyaviruses, it is necessary to establish a standardized approach to determine their molecular, antigenic, and pathogenic characteristics. Investigating the molecular and antigenic relationships between pathogenic and non-pathogenic viruses could help identify the determinants of disease. The hypothesised relationships could be investigated in experimental infection trials. Pathogenicity trials are possible for SGVs because a laboratory infection model has already been established in chicken embryos [95,96,97,98]. The suitability of the chicken embryo model is best demonstrated by the embryo pathology described in a comparison between Schmallenberg and Akabane [99]. Embryo mortality and pathological findings were akin to natural Schmallenberg virus and Akabane virus infections in ruminants [99]. Techniques in molecular epidemiology could complement traditional virological methods to help determine the threat posed by the Shamonda-like isolate detected during surveillance in the Australian orthobunyavirus ecosystem.

The type of diagnostic tests utilised have also evolved with the advent of molecular technology, and this has resulted in some challenges when comparing the early literature with the more recent Schmallenberg virus epidemic. A range of agent and antibody tests and sample types have been utilised to diagnose SGVs as the cause of congenital diseases. The detection of an SGV in a foetus with congenital disease provides a strong association between the virus and its role as an aetiological agent. However, as foetal immunocompetence develops, the virus may be cleared as the foetus becomes able to recognise and respond to pathogens [25]. As a result, the causative virus may not be isolated or detectable within foetal tissues [60]. Consequently, a negative virus culture or PCR result does not rule out an SGV infection as the cause of a disease. This is especially the case for cattle given the longer gestation period in comparison with smaller ruminant species (Table 2). As the ruminant placenta prevents the transfer of maternal antibodies during gestation [25], antibodies detected in a foetus are presumed to be the outcome of in utero infection [100]. Serological tests on the blood of a stillborn or aborted neonate can support a definitive diagnosis of congenital disease caused by SGVs that are known foetal pathogens. In the absence of foetal samples, blood from the associated dam should be submitted for testing to provide supportive evidence of previous viral infection.

The usefulness of blood is reflected in this review as it is the most common sample type utilised in both diagnostic test evaluation and monitoring surveys. Blood is necessary to detect SGVs in transmission, but the window to detect viraemia is short-lived [92,93]. Viruses may be isolated from blood in a window of 1–4 days in the 1–6 days following experimental infection [93,101,102]. In PCR testing, this window of detection for viraemia may be up to 8 days post-infection [103]. Meanwhile, following viraemia, neutralizing antibodies develop within a few days and may be detected for up to three years or longer [40,97,104]. The prolonged presence of antibodies following infection complicates the interpretation of serological tests based on a single sample from adult animals. This is because the antibodies detected may not indicate recent infection but rather exposure during previous transmission seasons. This is also complicated when animals may be infected with more than one orthobunyavirus in the Simbu group. It is difficult to distinguish the different viruses within the serogroup using serological tests because SGVs share antigenic similarities [105]. Further, the false-positive results for the virus-specific SBV-S PCR demonstrate that agent detection tests also struggle to distinguish between SGVs [65]. However, using a broadly or cross-reactive test allows for the detection of a virus at the serogroup level, including potentially previously unrecognised SGVs.

The commercially available ELISAs (Table 4) and the virus-specific SBV-S PCR are cross-reactive tests, but it is important to note that this detection does not necessarily reflect the risks or likelihood of disease. In regions where there are multiple SGVs in transmission, assays that can distinguish the individual SGVs are necessary in outbreak investigations to correctly identify the specific SGVs of veterinary importance. Currently, additional test methods are utilised following the detection of an SGV to identify the aetiological agent. This additional testing includes the use of specific VNTs [105,106]. However, even with VNTs, it can be difficult to differentiate between antibody titres and identify the specific SGV. This can occur when the same sample returns similar antibody titres across multiple specific VNTs [106]. Novel reagents and technologies could be investigated to develop a more efficient test method that allows for the rapid detection of specific SGVs. This includes utilising the Gc protein in ELISAs and investigating tools that allow for the simultaneous testing of antibodies against multiple proteins, such as the Luminex MAGPIX^®^ system [66,107]. These new diagnostic tests must be validated appropriately, using known positive and negative reference samples that encompass all SGVs.

Surveillance programs monitoring SGV transmission can also help provide supportive evidence in diagnostic investigations. Seroprevalence surveys were identified as the main method to determine the geographical distribution of SGVs in livestock. The distribution of SGVs may be monitored by collecting a serum sample at a single time point from wildlife [108,109,110] or animals held at a saleyard [111], or even by opportunistically using samples collected during control programs for other animal pathogens [111]. However, because of the persistence of antibodies, serosurveys sampled at a single time point, without regard to the age or location of the sampled animal, are not a real-time representation of virus transmission and limit interpretation of the data over a defined time interval. This is particularly evident in the high level of variability observed in the reported seroprevalence by location, host species sampled, and virus of interest (Supplementary Figure S1). As a result, the mean seroprevalence (Table 1) is presented to help provide rough estimates for sample size calculations for future surveys. The seasonal transmission of SGVs can be monitored through active surveillance programs that use a network of sentinel herds [102,112,113,114,115]. To effectively monitor SGV distribution using a sentinel network, it is critical to consider seasonal variations and geoclimatic regions when selecting locations for herds [19]. For a robust surveillance system, these active programs should be supplemented with the strategic testing of diagnostic submissions.

This review provides the justification to include strategic testing for cases of neurological disease in livestock. If a pathological SGV was to emerge in a naïve animal population, it could be anticipated that this disease presentation could precede reports of congenital malformations. This is exemplified by the difference in seasonal observations with Shuni virus. The acute onset of neurological disease in horses follows infection during times of high vector activity, resulting in disease primarily being reported in summer and autumn [16]. In contrast, for the SGVs that cause congenital malformations in ruminants, there is an interval between infection and parturition, with disease more likely to be observed in autumn or winter. Infections are more likely to occur in young animals because of a lack of acquired immunity in previous transmission seasons. Neurological disease in the post-natal period was observed preceding observations of congenitally malformed calves and lambs with Akabane virus [34,35,40,41]. Young horses were also more often reported with neurological signs following Shuni virus infection [44]. All animals showing consistent clinical neurological signs during favourable seasonal conditions for virus transmission should be investigated to ensure SGV-associated disease is not missed.

While rare, horses and adult cattle can develop a fatal neurological disease following infection with Shuni virus or Akabane virus [16,34,44,45,46]. Diagnoses of neurological signs associated with SGVs could be missed because disease would occur following acute infection. This means that serological tests may not be able to diagnose disease because antibodies may not yet be present. Given the viral tropism for nervous tissue, the brain is the obvious organ of choice for PCR testing, but the collection of brain tissue requires necropsy. This sample type would also be problematic to collect in regions with other viruses of high zoonotic consequences, such as for horses in Australia with concerns of Hendra virus (*Henipavirus hendraense*). In these circumstances, agent detection using blood or cerebral spinal fluid may present an alternative diagnostic approach. This approach was successful in retrospect, confirming viral infection with Shuni virus in horses with a consistent clinical presentation [16]. Alternatively, demonstrating seroconversion would confirm a recent infection. This strategic testing of appropriate diagnostic cases aids passive surveillance to help strengthen the existing monitoring of SGVs.

Publication bias and/or a lack of surveillance activity could result in gaps in the distribution and diversity of SGVs. This could explain the apparent absence of Clade B SGVs in Southeast Asia and North America (Figure 1). The absence of a Clade B SGV in Southeast Asia is unlikely. Recently, antibodies against Schmallenberg virus were detected in sera collected from small ruminants in Malaysia [116]. However, because the commercial ELISA utilised in this study was designed based on the N protein, the antibodies could not be distinguished from other SGVs. Nonetheless, the absence of a Clade B virus does not appear likely in Southeast Asia given that neighbouring regions have reported SGVs from both clades. Given the geographical isolation, the observations of an absence of a Clade B SGV in North America may be valid. However, this is difficult to evaluate because no publications of surveillance strategies from North America were assessed in this review. Additionally, the presence of a distantly related orthobunyavirus, Cache Valley virus (*Orthobunyavirus cacheense*), which also causes congenital defects in ruminants, could mask the emergence of a teratogenic SGV [117].

Restrictions applied during the systematic screening process of this scoping review led to several limitations. Publications were screened to include only original research in the English language with relevance to livestock species of interest. The restriction to English-language publications meant that a study on the experimental infection of pregnant pigs with Akabane virus (Supplementary Table S1) that was written in Japanese and an investigation of swine as a reservoir for Schmallenberg virus in French were not included [118,119]. In applying restrictions to original research, this review only evaluated assays with established development or validation pathways. This meant that diagnostic assays that have no evaluation pathways, like virus isolation and genomic sequencing, were not included. These methods are critical virological techniques and are essential when the virus of interest may be unknown. Finally, the focus on livestock species resulted in publications describing vector distribution being excluded. As arboviruses, vectors play an important role in the epidemiology of diseases caused by SGVs [120,121]. While reviewing the identified literature on *Culidoides* spp., midges were identified as a sample type for diagnostic testing and surveillance programs and their relevance and significance in these areas cannot be ignored.

It was also surprising that the field of molecular epidemiology and its associated tools were not identified during the review of surveillance methods despite their tremendous growth in the last decade. This could be because the allocation to a theme within this review was based on the study objectives of animal health impacts, diagnostic tests, or surveillance strategies. This focus meant that the details on the molecular characterisation of SGVs in the publications identified by our search strategy were not extracted for analysis. This is a significant limitation in this review, as molecular epidemiology allows for a better understanding of the distribution and regionalization of SGVs and how new viruses may have evolved in a particular region.

## 5. Conclusions

The Simbu group of viruses may cause severe livestock diseases, which usually manifest as congenital malformations, reproductive loss, and neurological disease. Although there can be a range of clinical presentations, clusters of congenital malformations, characterised by arthrogryposis and hydranencephaly, should raise a high level of suspicion of the involvement of an SGV. Among the 33 named orthobunyaviruses in the SGV subgroup, 6 have been identified to cause animal diseases: Akabane virus, Schmallenberg virus, Aino virus, Shuni virus, Peaton virus, and Shamonda virus. Disease diagnosis is complicated by the timing of foetal immunocompetence and test cross-reactivity. To demonstrate assay specificity, tests for both agents and antibodies should be validated with reference samples that encompass all of the SGVs. While broadly reactive tests are available to detect SGVs, novel diagnostic tools should be investigated so that individual viruses may be identified in a rapid and cost-effective manner. Tests must also be validated for surveillance purposes, as serosurveys remain the mainstay of monitoring methods, and an understanding of the spatial–temporal transmission must rely heavily on the longitudinal sampling of sentinel herds. Finally, further research is required to help determine the disease risk of novel orthobunyaviruses and how they could challenge current diagnostic and surveillance capabilities.

## Figures and Tables

**Table 1:** The number of records for viruses in the Simbu group extracted from the 224 publications identified for the scoping review protocol. A summary of the key animal health impacts, diagnostic assays, and monitoring methods is also provided.

Clade	Virus Species	Virus Names	Animal Health			Diagnostic Tests			Distribution			Total Records by Virus
			No. of Records	Species	Key Disease Syndrome	No. of Records	Highest Level of WOAH Validation	Validated Assay Types	No. of Records	Mean Seroprevalence ^1^	95% CI	
A	*Orthobunyavirus buttonwillowense*	Buttonwillow virus										
A	*Orthobunyavirus catqueense*	Cat Que virus							1	60	50–70	1
		Oya virus										
A	*Orthobunyavirus faceyense*	Facey’s paddock virus				1	No validation		1	-	-	2
A	*Orthobunyavirus ingwavumaense*	Ingwavuma virus	1	Porcine	No disease	1	Stage 1	PCR				2
A	*Orthobunyavirus jatobalense*	Jatobal virus										
A	*Orthobunyavirus manzanillaense*	Manzanilla virus										
		Inini virus										
A	*Orthobunyavirus mermetense*	Mermet virus										
A	*Orthobunyavirus oropoucheense*	Oropouche virus				1	Stage 1	PCR				1
		Iquitos virus										
		Madre de Dios virus										
		Perdoes virus										
		Pintupo virus										
A	*Orthobunyavirus thimiriense*	Thimiri virus				2	Stage 1	PCR	3	0	0	5
A	*Orthobunyavirus utingaense*	Utinga virus										
		Utive virus										
N/A	*Orthobunyavirus oyoense*	Oyo virus										
B	*Orthobunyavirus ainoense*	Aino virus	8	Bovine	Congenital malformations, neurological syndrome, reproductive disease	8	Stage 1	ELISA, PCR	21	27.9	20.4–35.5	37
B	*Orthobunyavirus akabaneense*	Akabane virus	43	Bovine, Ovine, Caprine	Congenital malformations, neurological syndrome, reproductive disease,	20	Stage 2	ELISA, PCR	32	23.5	14.6–32.4	95
				Porcine	No disease							
		Tinaroo virus	1	Bovine	No disease	3	Stage 1	PCR	5	24.6	20.9–28.3	9
		Yaba-7 virus										
B	*Orthobunyavirus schmallenbergense*	Schmallenberg virus	36	Bovine, Ovine, Caprine	Reproductive disease, congenital malformations, non-specific and gastrointestinal	21	Stage 2	ELISA, PCR, VNT, LAMP, RPA, IFA	51	34.9	27.0–42.8	108
				Porcine	No disease							
		Shamonda virus	2	Bovine, Ovine, Caprine	Congenital malformations	4	Stage 1	PCR	4	34.1	3.1–65.1	10
		Douglas virus	2	Bovine	No disease	3	Stage 1	PCR	5	14.0	8.8–19.3	10
		Sathuperi virus				4			3	34.9	8.2–61.5	7
B	*Orthobunyavirus peachesterense*	Peaton virus	3	Bovine	Congenital malformations, reproductive disease	5	Stage 1	PCR	9	21.6	15.7–27.5	17
B	*Orthobunyavirus saboense*	Sabo virus				3	Stage 1	PCR	1	6.6	0–13.9	4
B	*Orthobunyavirus saboense*	Sango virus	1	Ovine, Caprine	No disease	2	Stage 1	PCR				3
B	*Orthobunyavirus shuniense*	Shuni virus	6	Bovine, Equine	Neurological, congenital malformations, reproductive	4	Stage 2	ELISA, PCR				10
		Kaikalur virus										
B	*Orthobunyavirus simbuense*	Simbu virus				3	Stage 1	PCR	1	9.5	1.9–17.1	4
		Para virus										

^1^ This is a rough estimate of the mean seroprevalence, calculated by summarising the seroprevalence reported across publications. This estimate does not account for host or environmental factors such as animal species, age, location, or time of year.

**Table 2:** Congenital malformations and foetal pathology caused by Simbu group viruses during in utero development.

Gestational Stage at Infection	Species		
	Bovine	Ovine	Caprine
Pre-implantation			Embryonic death
Post-implantation	Hydranencephaly Porencephaly Myositis of skeletal muscles Arthrogryposis Hydranencephaly Porencephaly Stillborn	Arthrogryposis Brachygnathism Brain agenesis Hydranencephaly Lung hypoplasia Microencephaly Muscle neuronal atrophy and degeneration Porencephaly Scoliosis Spinal cord agenesis or hypoplasia	Foetal death
Following development of foetal immunocompetence	Arthrogryposis Stillborn Degenerative encephalopathy Clinical encephalopathy Stillborn		
References	[22,23,24,25,26,27]	[28,29,30,31]	[32,33]

**Table 3:** The frequency of clinical signs and necropsy findings for each virus in the Simbu group recognised as a cause of animal disease.

	Akabane Virus	Schmallenberg Virus	Shuni Virus	Aino Virus	Shamonda Virus	Peaton Virus
Total Cases	1675	553	48	17	15	2
Clinical signs	Congenital malformations: Unable to suckle, 140 Arthrogryposis, 118 Blind, 109 Scoliosis/vertebral deformities, 51 Brachgnathia inferior/prognathism, 45 Torticollis, 37 Skeletal muscle atrophy, 19 Lameness, 13 Leg extension, 11 Weak, 9 Deaf, 7 Cleft palate, 3 Dwarf, 2 Cryptorchid, 1	Congenital malformations: Arthrogryposis, 109 Scoliosis/Vertebral deformities, 82 Brachgnathia inferior/prognathism, 63 Torticollis, 46 Skeletal muscle atrophy, 1 Blind, 1 Cleft palate, 1	Congenital malformations: Arthrogryposis, 1 Torticollis, 1	Congenital malformations: Arthrogryposis, 13 Torticollis, 1 Leg extension, 7	Congenital malformations: Arthrogryposis, 12 Scoliosis/vertebral deformities, 11 Torticollis, 10	Congenital malformations: Arthrogryposis, 1 Scoliosis/vertebral deformities, 1 Blind, 1
	Neurological Astasia/Dysstasia, 90 Dull, 47 Recumbency, 31 Convulsions/tremors, 24 Circling, 15 Nystagmus, 15 Hypersensitivity, 14 Collison with barrier, 9 Paralysis, 8 Pupillary contraction, 4 Altered consciousness, 2	Neurological (only in animals surviving in-utero infection): Dull, 1 Proprioception deficits, 1, Strabismus, 1	Neurological: Ataxia, 10 Recumbency, 7 Paralysis, 5 Convulsions/tremors, 2 Hypersensitivity, 1 Circling, 1 Weak, 1	Neurological (only in animals surviving in utero infection): Ataxia, 8 Astasia/dysstasia, 8 Recumbency, 7 Hypersensitivity, 7 Circling, 7 Nystagmus, 1		
	Reproductive: Abortion, 76 Dystocia, 33 Perinatal mortality, 4 Mummified/autolyzed foetus, 2 Stillborn, 2	Reproductive: Abortion, 50 Mummified/autolyzed foetus, 16 Perinatal mortality, 10 Stillborn, 2	Reproductive: Abortion, 3	Reproductive Mummified/autolyzed foetus, 7 Stillborn, 4 Abortion, 1		Reproductive: Stillborn, 1
	Non-specific: Tachypnoea,47 Pyrexia,46 Ataxia, 44 Tachycardia, 42 Salivation, 25 Reduced appetite, 13 Hypothermia, 4 Jaundice, 1	Non-specific: Temporary decrease in milk yield, 350 Pyrexia, 227	Non-specific: Found dead, 12 Pyrexia, 9 Reduced appetite, 5 Anaemia, 2 Leukopenia, 2	Non-specific: Pyrexia, 7 Reduced appetite, 7		
	Gastrointestinal: Diarrhoea, 3	Gastrointestinal: Diarrhoea, 323				
Necropsy findings	Congenital malformations: Micro-/hydranencephaly, 190 Hydrocephalus/porencephaly, 90 Hypoplasia of the cerebellum, 56 Hypoplasia of the spinal cord, 55 Vestigial lungs/pulmonary hypoplasia, 25 Hypoplasia of the cerebrum, 3	Congenital malformations: Hypoplasia of the cerebellum, 86 Hypoplasia of the spinal cord, 67 Hydrocephalus/porencephaly, 45 Micro-/hydranencephaly, 41 Hypoplasia of the cerebrum, 12 Cardiac ventricular septal defect, 3 Vestigial lungs/pulmonary hypoplasia, 1 Colonic atresia, 1 Ectopic cordis, 1 Unilateral hydronephrosis, 1	No reports	Congenital malformations: Micro-/hydranencephaly, 6 Hypoplasia of the cerebellum, 3	Congenital malformations: Micro-/hydranencephaly, 2 Hypoplasia of the cerebrum, 1	Congenital malformations: Hydrocephalus/porencephaly, 2 Hypoplasia of the cerebellum, 1

**Table 4:** Details of six commercially available ELISAs for Akabane virus or Schmallenberg virus. The positive and negative agreement percentages were obtained with samples of a known status by comparison studies using a virus neutralisation test as the relative gold standard.

ELISA	Format	Target (Virus)	Sample	Sample Status and Size	Positive Agreement (%)	Negative Agreement (%)	Reference
ID-Screen Akabane ELISA (Innovative Diagnostics, Grabels, France)	Competition	Whole virus (Akabane virus)	Serum	Positive (n = 378) Negative (n = 334)	96.0–98.9	99.7	[71]
			Serum	Positive (n = 153) Negative (n = 537)	93.5	82.3	[72]
Akabane ELISA kit (Chisso Corp, Yokohama)	Competition	Gc glycoprotein (Akabane virus)	Serum	Positive (n = 378) Negative (n = 334)	78.0	100	[71]
ID-Screen Schmallenberg Virus Multi-Species ELISA (Innovative Diagnostics, Grabels, France)	Competition	Nucleoprotein (Schmallenberg virus)	Serum	Positive (n = 45) Negative (n = 45)	96–100	100	[73]
ID-Screen Schmallenberg Virus Indirect Multi-Species ELISA (Innovative Diagnostics, Grabels, France)	Indirect	Nucleoprotein (Schmallenberg virus)	Serum	Positive (n = 180) Negative (n = 1364)	97.2	99.8	[74]
IDEXX Schmallenberg Virus Antibody Test Kit (IDEXX, Hoofddorp, Netherlands)	Indirect	Nucleoprotein (Schmallenberg virus)	Serum	Positive (n = 45) Negative (n = 45)	78–93	93–98	[73]
			Serum	Positive (n = 153) Negative (n = 537)	80.4	93.5	[72]
Svanovir Schmallenberg Antibody ELISA (Svanova, Uppsala, Sweden)	Indirect	Whole virus (Schmallenberg virus)	Serum	Positive (n = 82) Negative (n = 6)	94	50	[75]
			Bulk milk	Positive (n = 82) Negative (n = 6)	98	100	

## Supplementary Materials

The following supporting information can be downloaded at https://www.mdpi.com/article/10.3390/v16020294/s1, Supplementary Table S1: The distribution, insect vector, host range, and disease observations for the 33 viruses within the Simbu serogroup as arranged by their species designation; Supplementary Table S2: Data extracted from eligible publications in a scoping review to identify veterinary-important Simbu group viruses. This information was used to produce a summary of the associated disease presentations, monitoring methods, and diagnostic assays of the SGVs with veterinary importance; Supplementary Table S3: Histopathological details extracted from publications describing the animal health outcomes following natural or experimental infection of a Simbu group virus in livestock; Supplementary Table S4: PCR details extracted from publications describing the molecular assays developed and validated for the detection of Simbu group viruses; Supplementary Figure S1: Mean seroprevalence extracted from seroprevalence studies describing the distribution and diversity of SGVs by (A) country, (B) host species, and (C) virus. Supplementary note: The two search strategies used to find eligible publications across the five databases. The repRows function was used to match variables entered as a comma-separated list and extract the details into multiple records for the same publication.
