# Supplementary material for: Revisiting the Importance of Orthobunyaviruses for Animal Health: A Scoping Review of Livestock Disease, Diagnostic Tests, and Surveillance Strategies for the Simbu Serogroup
Source: Viruses. 2024 Feb 15;16(2):294. doi: 10.3390/v16020294 (PMC10892073; doi:10.3390/v16020294)
Supplement: Supplementary file 1 [file viruses-16-00294-s001.zip › Supplementary note.pdf]

1. (“Bunyavir*” OR “orthobunyavirus*”) AND (“Shamonda*” OR “Simbu*”)

2. (“Bunyavir*” OR “*orthobunyavirus*” OR “Akabane” OR “Tinaroo” OR “Yaba-7” OR “Jatobal” OR “Buttonwillow” OR “Cat Que” OR “Ingwavuma” OR “Inini” OR “Manzanilla” OR “Mermet” OR “Facey's paddock” OR “Iquitos” OR “Madre de Dios” OR “Oropouche” OR “Perdoes” OR “Pintupo” OR “Utinga” OR “Utive” OR “Douglas” OR “Sango” OR “Sathuperi” OR “Peaton” OR “Schmallenberg” OR “Shamonda” OR “Sabo” OR “Aino” OR “Kaikalur” OR “Shuni” OR “Oya” OR “Para” OR “Simbu” OR “Thimiri”) AND (“bovine” OR “cattle” OR “cow” OR “calf” OR “calves” OR “dairy” OR “beef” OR “ovine” OR “sheep” OR “caprine” OR “goat” OR “equine” OR “horse” OR “porcine” OR “pig” or “pork”)
