# Supplementary material for: Revisiting the Importance of Orthobunyaviruses for Animal Health: A Scoping Review of Livestock Disease, Diagnostic Tests, and Surveillance Strategies for the Simbu Serogroup
Source: Viruses. 2024 Feb 15;16(2):294. doi: 10.3390/v16020294 (PMC10892073; doi:10.3390/v16020294)
Supplement: Supplementary file 1 [file viruses-16-00294-s001.zip › Supplementary Table S1.pdf]

Supplementary Table S1. The distribution, insect vector, host range, and disease observations for the 33 viruses within the Simbu serogroup as arranged by their species designation [1]

**Virus species**	**Virus name**	**Clade**	**Accession**	**Distribution**	**Insect vectors**	**Animal hosts**	**Animal disease**<br>**(Species, clinical signs, and pathological description)**
Orthobunyavirus ainoense	Aino virus	B	L: HE795087<br>M: HE795088<br>S: HE795089	Australia [2], Israel [3], Japan [4], South Korea [5], Tanzania [6]	*Culicodes* midges, mosquitoes [4]	Bovine [2, 7, 8], Caprine [9], Cervids [10], Equine [11, 12], Ovine [9], Swine [8, 12]	Bovine – astasia [13], leukopenia [13], congenital malformations following intrauterine infection [14-20], disseminated non-purulent encephalomyelitis following experimental infection [21]<br><br>Chicken embryo – death and deformities [22]
Orthobunyavirus akabaneense	Akabane virus	B	L: AB190458<br>M: AB100604<br>S: AB000851	Australia [23], China [24], Cyprus [25], Oman [26], Iran [27], Israel [28], Japan [29], Kenya [30], South Africa [31], South Korea [32], Taiwan [33], Tanzania [6], Vietnam [34]	*Culicodes* midges [31]	Bovine [35], Caprine [35], Cervid [36], Equine [36, 37], Ovine [35], *Rhinocerotidae* (Black and White Rhino) [38], Swine [39]	Bovine, caprine and ovine - congenital malformations following intrauterine infection [35, 40, 41]<br>Bovine – non-suppurative encephalitis, abortion [33]<br><br>Porcine – decreased appetite, pyrexia, leukopenia, foetal infection [39]<br><br>Chicken embryo – death and deformities [22]
	Tinaroo virus	B	L: MH484339<br>M: MH484340<br>S: MH484341	Australia [42], Tanzania [6]	*Culicodes* midges [42]	Bovine [36], Caprine [36], Ovine [36]	Chicken embryo – death and deformities [22]
	Yaba-7 virus	unassigned	L: MH484348<br>M: MH484349<br>S: MH484350	Nigeria [43]	Mosquitoes [43]	Bovine [43]	No reports
Orthobunyavirus buttonwillowense	Buttonwillow virus	A	L: KF697160<br>M: KF697161<br>S: KF697162	Canada [44], United States of America [44]	*Culicodes* midges, mosquitoes [44]	Laprine (rabbits and hares) [44], *Sciuridae* (marmot) [44]	Murine – subclinical after subcutaneous or intraperitoneal injection. Death following intracranial inoculation [44]<br><br>Laprine and ovine – asymptomatic [44]
Orthobunyavirus catqueense	Cát Quế virus	A	L: JQ675598<br>M: JQ675599<br>S: JQ675600	China [45], India [46], Uganda [47], Vietnam [34]	Mosquitoes [34]	Swine [34]	Murine – paralysis, death [46]<br><br>Human - paediatric encephalitis [34]
	Oya virus	unassigned	L: JX983194<br>M: JX983193<br>S: JX983192	China [48], India [49], Malaysia [50], Vietnam [34]	Mosquitoes [34]	Swine [50]	No reports
Orthobunyavirus faceyense	Facey's paddock virus	A	L: KF697138<br>M: KF697137<br>S: KF697136	Australia [51]	Mosquitoes [51]	Not known [22]	Chicken embryo – death and deformities [22]

**Virus species**	**Virus name**	**Clade**	**Accession**	**Distribution**	**Insect vectors**	**Animal hosts**	**Animal disease (Species, clinical signs, and pathological description)**
Orthobunyavirus ingwavumaense	Ingwavuma virus	A	L: KF697139<br>M: KF697140<br>S: KF697141	India [52], Indonesia [53], Nigeria [54], South Africa [55], Thailand [56]	Mosquitoes [55]	Avian [55], Bovine [53], Canine [56], Caprine [53], Human [54], Ovine [53], Swine [56]	No reports
Orthobunyavirus jatobalense	Jatobal virus	A	L: JQ675603<br>M: JQ675602<br>S: JQ675601	Brazil [57]	No reports	Procyonidae (coati) [57]	No reports
Orthobunyavirus manzanillaense	Manzanilla virus	A	L: KF697150<br>M: KF697149<br>S: KF697148	Trinidad [58]	Mosquitoes [58]	*Atelidae* (howler monkey) [58], *Molossidae* (black mastiff bat) [59]	No reports
	Inini virus	A	No reports	French Guiana [60]	No reports	Avian (toucan) [60]	No reports
Orthobunyavirus mermetense	Mermet virus	A	L: KF697153<br>M: KF697151<br>S: KF697152	Guatemala [61], United States of America [62]	Mosquitoes [63]	Avian (warbler, purple martin, black-bird, thrush, blue jay) [64]	Murine- newborn mice illness following intracerebral inoculation [65]
Orthobunyavirus oropoucheense	Oropouche virus	A	L: AF484424<br>M: AF441119<br>S: AY237111	Brazil [66], Peru [67], Trinidad [68]	*Culicodes* midges [69], mosquitoes [70]	Humans [68]	Human – febrile illness [66], laboratory-acquired infection [42].
	Iquitos virus	A	L: KF697142<br>M: KF697143<br>S: KF697144	Peru [71]	No reports	Humans [71]	Human – febrile illness [71]
	Madre de Dios virus	A	L: KF697147<br>M: KF697145<br>S: KF697146	Venezuela [72]	No reports	*Cebidae* (capuchin) [72]	Primates - an epizootic of illness among sylvatic monkeys (associated with reports of dead and moribund monkeys) [72]
	Perdões virus	A	L: KP691627<br>M: KP691628<br>S: KP691629	Brazil [73]	No reports	*Callitrichidae* (marmoset) [73]	No reports
	Pintupo virus	A	No reports	Panama [74]	No reports	Folivora – sloths [74]	No reports
Orthobunyavirus oyoense	Oyo virus	unassigned	L: HM639780<br>M: HM639779<br>S: HM639778	Nigeria [75]	No reports	No reports	No reports
Orthobunyavirus peachesterense	Peaton virus	B	L: HE795093<br>M: HE795094<br>S: HE795095	Australia [36], Israel [76], Japan [77], South Korea [78], Tanzania [6]	*Culicodes* midges [79]	Bovine [36], Caprine [36], Cervid [36], Equine [36], Ovine [36], Swine [80]	Bovine – congenital abnormalities and abnormal birth [76, 77]<br><br>Chicken embryo – death [22]
Orthobunyavirus saboense	Sabo virus	B	L: HE795096<br>M: HE795097<br>S: HE795098	Nigeria [43], Tanzania [6]	*Culicodes* midges [81]	Bovine [43], Caprine [43]	No reports
Orthobunyavirus saboense	Sango virus	B	L: HE795099<br>M: HE795100<br>S: HE795101	Nigeria [43]	*Culicodes* midges [81]	Bovine [43]	No reports

Virus species	Virus name	Clade	Accession	Distribution	Insect vectors	Animal hosts	Animal disease<br>(Species, clinical signs, and pathological description)
Orthobunyavirus schmallenbergense	Schmallenberg virus	B	L: KC355457<br>M: KC355458<br>S: KC355459	Albania [82], Austria [83], Belgium [84], China [85], Croatia [86], France [84], Germany [84], Greece [87], Iraq [88], Ireland [89], Italy [84], Kosovo [82], Luxembourg [84], Namibia [90], the Netherlands [84], Poland [91], Russia [92], Slovenia [93], Spain [84], Turkey [94], the United Kingdom [84], Tanzania [6]	*Culicodes* midges [95]	Bovine [96], Caprine [97], Cervids [98], Ovine [96], Ungulates [99]	Bovine, caprine and ovine - congenital malformations following intrauterine infection [100, 101], stillborn [100], premature birth [100]<br><br>Bovine - sudden decreased milk production, watery diarrhea and pyrexia [102]
	Douglas virus	B	L: HE795090<br>M: HE795091<br>S: HE795092	Australia [42], Papua New Guinea [36], Tanzania [6]	*Culicodes* midges [42]	Bovine [36], Caprine [36],<br>Cervid [36], Equine [36], Ovine [36]	Chicken embryo – death and deformities [22]
	Sathuperi virus	B	L: HE795102<br>M: HE795103<br>S: HE795104	India [103], Israel [3], Japan [104], Nigeria [43], South Korea [5], Tanzania [6]	*Culicodes* midges [81], mosquitoes [103]	Bovine [43]	No reports
	Shamonda virus	B	L: HE795105<br>M: HE795106<br>S: HE795107	Israel [3], Japan [105], Nigeria [43], Tanzania [6]	*Culicodes* midges [81]	Bovine [43]	Bovine - congenital malformations following intrauterine infection [106]
Orthobunyavirus shuniense	Shuni virus	B	L: KF153118<br>M: KF153117<br>S: KC510272	Israel [107], Nigeria [81], South Africa [108].	*Culicodes* midges [81]	Bovine [43], Equine [108], Human [43], Ovine [43]	Bovine – paralysis of the head and neck, ataxia, swallowing difficulties, dysstasia [107]<br><br>Equine – neurological disease: ataxia, tremors, convulsions, recumbency, quadriplegia, anorexia, leukopenia, depression, anaemia, icterus, hyperaesthesia [108].<br><br>Human – febrile illness, laboratory-acquired infection [109]
	Kaikalur virus	unassigned	L: MH484297<br>M: MH484298<br>S: MH484299	India [110]	Mosquitoes [110]	No reports	No reports
Orthobunyavirus simbuense	Simbu virus	B	L: HE795108<br>M: HE795109<br>S: HE795110	South Africa [111], Tanzania [6]	Mosquitoes [111]	No reports	No reports
	Para virus	unassigned	No reports	Argentina [112]	Mosquitoes [112]	No reports	No reports
Orthobunyavirus thimiriense	Thimiri virus	A	L: MH484336<br>M: MH484337<br>S: MH484338	India [113], Australia [36]	*Culicodes* midges [114]	Avian - *Ardeola grayii* (Indian pond heron) [113]	Chicken embryo – death [22]

Virus species	Virus name	Clade	Accession	Distribution	Insect vectors	Animal hosts	Animal disease (Species, clinical signs, and pathological description)
Orthobunyavirus utingaense	Utinga virus	A	L: KF697154<br>M: KF697155<br>S: KF697156	Brazil [115]	No reports	*Folivora* - sloths [115]	No reports
	Utive virus	A	L: KF697157<br>M: KF697159<br>S: KF697158	Panama [74]	No reports	*Folivora* – sloths [74]	No reports

1. Abudurexiti A, Adkins S, Alioto D, Alkhovsky SV, Avšič-Županc T, Ballinger MJ, et al. Taxonomy of the order Bunyavirales: update 2019. Arch Virol. 2019;164(7):1949-65. Epub 2019/05/09. doi: 10.1007/s00705-019-04253-6. PubMed PMID: 31065850; PubMed Central PMCID: PMCPMC6641860.
2. Cybinski DH, George TDS. A survey of antibody to Aino virus in cattle and other species in Australia. Australian Veterinary Journal. 1978;54(8):371-3. doi: 10.1111/j.1751-0813.1978.tb02504.x. PubMed Central PMCID: PMC718555.
3. Brenner J, Yanase T, Kato T, Yaakobi S, Khinich E, Paz R, et al. Serological evidence suggests that several simbu serogroup viruses circulated in israel. Veterinaria Italiana. 2019;55(1):81-9. doi: 10.12834/VetIt.1397.7622.2. PubMed Central PMCID: PMC30951185.
4. Takahashi K, Oya A, Okada T, Matsuo R, Kuma M, Noguchi H. Aino virus, a new member of Simbu group of arbovirus from mosquitoes in Japan. Japanese Journal of Medical Science and Biology. 1968;21(2):95-101 pp. doi: 10.7883/yoken1952.21.95. PubMed PMID: CABI:19701001610.
5. Jun K, Yanaka T, Lee K, Lee J. Seroprevalence of bovine arboviruses belonging to genus Orthobunyavirus in South Korea. Journal of Veterinary Medical Science. 2018;80(10):1619-23. doi: 10.1292/jvms.17-0542. PubMed PMID: CABI:20193031646.
6. Mathew C, Klevar S, Elbers AR, van der Poel WH, Kirkland PD, Godfroid J, et al. Detection of serum neutralizing antibodies to Simbu sero-group viruses in cattle in Tanzania. 2015;1:208.
7. Cho JJ, Shin YK, Yoon S, Yoon SS, Lee O, Song JY, et al. Seroepidemiological studies of aino virus infection in Korean cattle. Journal of Bacteriology and Virology. 2009;39(4):345-51. doi: 10.4167/jbv.2009.39.4.345.
8. Seong IL, Chang HK, Dong ST, Seong HK, Dong KY. Sero-survey on Aino, Akabane, Chuzan, bovine ephemeral fever and Japanese encephalitis virus of cattle and swine in Korea. Journal of Veterinary Science. 2007;8(1):45-9. doi: 10.4142/jvs.2007.8.1.45.
9. Hwang J-M, Ga YJ, Yeh J-Y. Factors associated with seropositivity to Aino virus among sheep and goats in South Korea. Acta veterinaria Hungarica. 2022. doi: 10.1556/004.2022.00015. PubMed PMID: MEDLINE:35895542.
10. Yeh JY, Ga YJ. Seroepidemiology of Aino Virus in Farmed and Free-Ranging Cervids in the Republic of Korea. Frontiers in Veterinary Science. 2021;8. doi: 10.3389/fvets.2021.702978.
11. Yang DK, Kim BH, Kweon CH, Nah JJ, Kim HJ, Lee KW, et al. Serosurveillance for Japanese encephalitis, Akabane, and aino viruses for Thoroughbred horses in Korea. Journal of Veterinary Science. 2008;9(4):381-5. doi: 10.4142/jvs.2008.9.4.381.
12. Fukuyoshi S, Takehara Y, Takahashi K, Mori R. The incidence of antibody to Aino virus in animals and humans in Fukuoka. 1981;1(1):41-3.
13. Ishibashi K, Asada K, Shirakawa H, Nakamura H, Watanabe A. Isolation of Aino virus from a cow with astasia and leukopenia. Journal of the Japan Veterinary Medical Association. 1992;45(11):837-40. PubMed PMID: CABI:19932278477.
14. Tsuda T, Yoshida K, Ohashi S, Yanase T, Sueyoshi M, Kamimura S, et al. Arthrogryposis, hydranencephaly and cerebellar hypoplasia syndrome in neonatal calves resulting from intrauterine infection with Aino virus. Veterinary Research. 2004;35(5):531-8. doi: 10.1051/vetres:2004029.
15. Uchinuno Y, Noda Y, Shirakawa H, Nagasue S, Nagano M, Ohe R. Congenital malformations in calves associated with Aino virus prevalence in Fukuoka Prefecture in 1995 to 1996. Journal of the Japan Veterinary Medical Association. 1997;50(12):709-12. PubMed PMID: CABI:19982209394.
16. Ishibashi K, Tomishita Y, Shirakawa H, Takaya M, Watanabe A. Congenital scoliosis in calves suspected of Aino virus infection. Journal of the Japan Veterinary Medical Association. 1994;47(2):87-90. PubMed PMID: CABI:19942206638.
17. Kitano Y, Yamashita S, Furukawa M, Makinoda K. Congenital abnormalities in calves suggesting Aino virus infection in Kagoshima Prefecture. Journal of the Japan Veterinary Medical Association. 1993;46(6):469-71. PubMed PMID: CABI:19932286937.

18. Fukutomi T, Ouchi M, Okuda K, Maruno F, Tabayashi K. Congenital abnormality in a calf apparently the result of an aino virus infection in Okayama prefecture. Journal of the Japan Veterinary Medical Association. 1991;44(1):17-9. PubMed PMID: CABI:19912253082.
19. Egashira T, Itimaru H, Yoshinaga N, Enaga N, Utikoshi N, Minamikawa R. Abnormal birth of cattle suggesting an Aino virus infection. Journal of the Japan Veterinary Medical Association. 1989;42(2):94-6. PubMed PMID: CABI:19902298901.
20. Coverdale OR, Cybinski DH, Stgeorge TD. CONGENITAL-ABNORMALITIES IN CALVES ASSOCIATED WITH AKABANE VIRUS AND AINO VIRUS. Australian Veterinary Journal. 1978;54(3):151-2. doi: 10.1111/j.1751-0813.1978.tb05538.x. PubMed PMID: WOS:A1978ES96400017.
21. Moriwaki M, Miura Y, Hayashi S, Ishitani R. Histopathological findings of calves infected experimentally with Aino virus. Natl Inst Anim Health Q (Tokyo). 1977;17(3):95-106. PubMed Central PMCID: PMC917129.
22. McPhee DA, Parsonson IM, Della-Porta AJ, Jarrett RG. Teratogenicity of Australian Simbu serogroup and some other Bunyaviridae viruses: the embryonated chicken egg as a model. Infection and Immunity. 1984;43(1):413-20. doi: 10.1128/iai.43.1.413-420.1984. PubMed PMID: CABI:19842239798.
23. Hartley WJ, Wanner RA, Della-Porta AJ, Snowdon WA. Serological evidence for the association of Akabane virus with epizootic bovine congenital arthrogryposis and hydranecephaly syndromes in New South Wales. 1975;51(2):103-4. doi: https://doi.org/10.1111/j.1751-0813.1975.tb09422.x.
24. Zhu P, Xiao L, Zhu J, Xie M, Gao L, Li H. Isolation and Identification of Akabane Virus from Cattle in China. Chinese Journal of Virology. 2021;37(2):382-8. PubMed PMID: CSCD:6934170.
25. Sellers RF, Herniman KAJ. Neutralising antibodies to Akabane virus in ruminants in Cyprus. Trop Anim Health Prod. 1981;13(1):57-60. doi: 10.1007/BF02237891. PubMed Central PMCID: PMC7256874.
26. Al-Busaidy SM, Mellor PS. Isolation and identification of arboviruses from the Sultanate of Oman. Epidemiology and Infection. 1991;106(2):403-13. doi: 10.1017/S095026880004855X. PubMed Central PMCID: PMC1850363.
27. Ahourai P, Gholami MR, Ezzi A, Kargar R, Khedmati K, Aslani A, et al. Bovine congenital arthrogryposis & hydranencephaly outbreaks attributed to Akabane virus infection in Iran. Archives de l'Institut Razi. 1992;(42/43):51-6. PubMed PMID: CABI:19952216125.
28. Shimshony S. An epizootic of Akabane disease in bovines, ovines and caprines in Israel, 1969-70: epidemiological assessment. Acta Morphologica Academiae Scientiarum Hungaricae. 1980;28(1-2):197-9.
29. Miura Y, Hayashi S, Ishihara T, Inaba Y, Omori T, Matumoto M. Neutralizing antibody against Akabane virus in precolostral sera from calves with congenital arthrogryposis-hydranencephaly syndrome. Brief report. Archiv fur die Gesamte Virusforschung. 1974;46(3/4):377-80. doi: 10.1007/bf01240082. PubMed PMID: CABI:19752283999.
30. Metselaar D, Robin Y. Akabane virus isolated in Kenya. Vet Rec. 1976;99(5):86. Epub 1976/07/31. doi: 10.1136/vr.99.5.86-a. PubMed PMID: 982784.
31. Theodoridis A, Nevill EM, Els HJ, Boshoff ST. Viruses isolated from Culicoides midges in South Africa during unsuccessful attempts to isolate bovine ephemeral fever virus. Onderstepoort Journal of Veterinary Research. 1979;46(4):191-8. PubMed Central PMCID: PMC551368.
32. Bak UB, Lim CH, Cheong CK, Hwang WS, Cho MR. Outbreaks of Akabane disease of cattle in Korea. Korean Journal of Veterinary Research. 1980;20(1):65-78. PubMed PMID: CABI:19802261614.
33. Liao YK, Lu YS, Goto Y, Inaba Y. The isolation of Akabane virus (Iriki strain) from calves in Taiwan. 1996;36(1):33-9. doi: https://doi.org/10.1002/jobm.3620360108.
34. BRYANT JE, CRABTREE MB, NAM VS, YEN NT, DUC HM, MILLER BR. ISOLATION OF ARBOVIRUSES FROM MOSQUITOES COLLECTED IN NORTHERN VIETNAM %J The American Journal of Tropical Medicine and Hygiene Am J Trop Med Hyg. 2005;73(2):470-3. doi: 10.4269/ajtmh.2005.73.470.

35. Inaba Y. Akabane Disease : An Epizootic Congenital Arthrogryposis-Hydranencephaly Syndrome in Cattle, Sheep and Goats Caused by Akabane Virus. Japan Agricultural Research Quarterly. 1979;13(2):123-33.
36. St George TD, Cybinski DH, Filippich C, Carley JG. The isolation of three Simbu group viruses new to Australia. Australian Journal of Experimental Biology and Medical Science. 1979;57(6):581-2. doi: 10.1038/icb.1979.60. PubMed PMID: CABI:19800577828.
37. Furuya Y, Shoji H, Inaba Y, Matumoto M. Antibodies to Akabane virus in horses, sheep and goats in Japan. Veterinary Microbiology. 1980;5(3):239-42. doi: 10.1016/0378-1135(80)90010-3.
38. Swanepoel H, Crafford J, Quan M. A scoping review of viral diseases in african ungulates. Veterinary Sciences. 2021;8(2):1-32. doi: 10.3390/vetsci8020017.
39. Maruyama S, Maruyama F, Tanaka Y, Itoh H, Uematsu N. Experimental infection of pigs with Akabane virus. Uirusu Journal of virology. 1983;33(2):131-3. doi: 10.2222/jsv.33.131.
40. Konno S. Pathology of Akabane disease, congenital abnormality in cattle. Japanese Journal of Animal Reproduction. 1977;22(5):xxxix-lv. PubMed PMID: CABI:19782228567.
41. Kurogi H, Inaba Y, Takahashi E, Sato K, Goto Y. Experimental infection of pregnant goats with Akabane virus. Natl Inst Anim Health Q (Tokyo). 1977;17(1):1-9.
42. Cybinski DH. Douglas and Tinaroo Viruses: Two Simbu Group Arboviruses Infecting Culicodes brevitarsis and Livestock in Australia. Australian Journal of Biological Sciences. 1984;37:91-7. PubMed PMID: CABI:19842245086.
43. Causey OR, Kemp GE, Causey CE, Lee VH. Isolations of Simbu-group viruses in Ibadan, Nigeria 1964–69, including the new types Sango, Shamonda, Sabo and Shuni. Annals of Tropical Medicine and Parasitology. 1972;66(3):357-62. doi: 10.1080/00034983.1972.11686835.
44. Reeves WC, Scrivani RP, Hardy JL, Roberts DR, Nelson RL. Buttonwillow virus, a new arbovirus isolated from mammals and Culicoides midges in Kern County, California. American Journal of Tropical Medicine and Hygiene. 1970;19(3):544-51 pp. doi: 10.4269/ajtmh.1970.19.544. PubMed PMID: CABI:19711000455.
45. Zhang J, Wang J, Wang L, Fu S, Li M, Zhao G, et al. Molecular Characterization and Seroprevalence in Pigs of SC0806, a Cat Que Virus Isolated from Mosquitoes in Sichuan Province, China. Vector-Borne and Zoonotic Diseases. 2015;15(7):423-31. doi: 10.1089/vbz.2014.1767. PubMed PMID: BIOSIS:PREV201600040995.
46. Whitmer SLM, Yadav PD, Prasad S, Chaubal GY, Francis A, Klena J, et al. Characterization of unknown orthobunya-like viruses from India. Viruses. 2018;10(9):451. doi: 10.3390/v10090451. PubMed PMID: CABI:20193023014.
47. Kumari S, Suman Y, Sanjit B, Sulochana K, Yadav JP, Samander K. The Cat Que Virus: a resurfacing orthobunyavirus could lead to epidemics. VirusDisease. 2021;32(4):635-41. doi: 10.1007/s13337-021-00745-9. PubMed PMID: CABI:20210490689.
48. Wu D, Zhang X, Zhang H, Tan Q, Zhou H, Wang H, et al. Detection and molecular characterization of the Akabane virus and Oya virus in Guangdong province, China. Chinese Journal of Virology. 2020;36(1):84-91. doi: 10.13242/j.cnki.bingduxuebao.003622. PubMed PMID: CABI:20210110740.
49. Yadav P, Shete A, Bondre V, Patil D, Kokate P, Chaudhari S, et al. Isolation and characterization of Oya virus a member of Simbu serogroup, family Bunyaviridae, isolated from Karnataka, India. Infection, Genetics and Evolution. 2016;44:122-6. doi: 10.1016/j.meegid.2016.06.049. PubMed Central PMCID: PMC27374486.
50. Kono Y, Yusnita Y, Mohd Ali AR, Maizan M, Sharifah SH, Fauzia O, et al. Characterization and identification of Oya virus, a Simbu serogroup virus of the genus Bunyavirus, isolated from a pig suspected of Nipah virus infection. Arch Virol. 2002;147(8):1623-30. Epub 2002/08/16. doi: 10.1007/s00705-002-0838-y. PubMed PMID: 12181680.
51. Doherty RL, Carley JG, Kay BH, Filippich C, Marks EN, Frazier CL. Isolation of virus strains from mosquitoes collected in Queensland Australia 1972-1976. Australian Journal of Experimental Biology

and Medical Science. 1979;57(5):509-20. doi: 10.1038/icb.1979.52. PubMed PMID: BIOSIS:PREV198171028304.
52. Yergolkar PN, Geevarghese G, Shaikh BH, Dandewate CN. Isolation of Ingwavuma virus from a sentinel pig in Kolar District, Karnataka, India. Virus Information Exchange Newsletter for South-East Asia and the Western Pacific. 1991;8(3):127. PubMed PMID: CABI:19920511153.
53. Converse JD, Tan RI, Rachman IT, Lee VH, Shope RE. Ingwavuma virus (Simbu group) from Culex and Mansonia mosquitoes (Diptera: Culicidae) in Indonesia. Journal of Medical Entomology. 1985;22(3):339-42. doi: 10.1093/jmedent/22.3.339. PubMed PMID: CABI:19860531043.
54. Fagbami A. Human arthrpod-borne virus infections in Nigeria - serological and virological investigations at Shaki, Oyo state. Journal of Hygiene Epidemiology Microbiology and Immunology. 1978;22(2):184-9. PubMed PMID: WOS:A1978GB28700007.
55. McLNtosh BM, Jupp PG, Sousa J. Further isolations of arboviruses from mosquitoes collected in Tongaland, South Africa, 1960-1968. Journal of Medical Entomology. 1972;9(2):155-9. doi: 10.1093/jmedent/9.2.155. PubMed PMID: CABI:19722703830.
56. Top Jr FH, Kraivapan C, Grossman RA, Rozmiarek H, Edelman R, Gould DJ. Ingwavuma virus in Thailand: infection of domestic pigs. American Journal of Tropical Medicine and Hygiene. 1974;23(2):251-7. doi: 10.4269/ajtmh.1974.23.251.
57. Figueiredo LT, Da Rosa AP. Jatobal virus antigenic characterization by ELISA and neutralization test using EIA as indicator, on tissue culture. Memórias do Instituto Oswaldo Cruz. 1988;83(2):161-4. doi: 10.1590/S0074-02761988000200003. PubMed Central PMCID: PMC2593820.
58. Anderson CR, Spence LP, Downs WG, Aitken THG. Manzanilla Virus: a New Virus Isolated from the Blood of a Howler Monkey in Trinidad, W. I. The American journal of tropical medicine and hygiene. 1960;9(1):78-80. doi: 10.4269/ajtmh.1960.9.78.
59. Price JL. Serological evidence of infection of Tacaribe virus and arboviruses in Trinidadian bats. American Journal of Tropical Medicine and Hygiene. 1978;27(1 I):162-7. doi: 10.4269/ajtmh.1978.27.162.
60. Report on the activity of the Pasteur Institute of French Guiana and the Territory of Inini during 19471948. 101 pp.- pp. p.
61. Gutierrezv E, Calisher CH, Maness KSC, Lord RD. Isolation of Mermet virus in Guatemala. Boletin De La Oficina Sanitaria Panamericana. 1975;79(1):24-9. PubMed PMID: WOS:A1975AK69500004.
62. Calisher CH, Kokernot RH, De Moore JF, Boyd KR, Hayes J, Chappell WA. Arbovirus studies in the Ohio-Mississippi Basin, 1964-1967. VI. Mermet: A Simbu-group arbovirus. The American journal of tropical medicine and hygiene. 1969;18(5):779-88. doi: 10.4269/ajtmh.1969.18.779.
63. Jakob WL, Francy DB, Trimble JM, Calisher CH. Recovery of mermet virus from Culex mosquitoes (Diptera: Culicidae) collected in Memphis, Tennessee, USA. Journal of Medical Entomology. 1979;16(1):80-1. doi: 10.1093/jmedent/16.1.80. PubMed Central PMCID: PMC522124.
64. Calisher CH, Ahmann SJ, Grimstad PR, Hamm JG, Parsons MA. Distribution and prevalence of Mermet virus infections in the central United States. 1981;1(2):473-6.
65. Rogers MB, Gulino KM, Tesh RB, Cui L, Fitch A, Unnasch TR, et al. Characterization of five unclassified orthobunyaviruses (Bunyaviridae) from Africa and the Americas. Journal of General Virology. 2017;98(9):2258-66. doi: 10.1099/jgv.0.000899. PubMed Central PMCID: PMC28885138.
66. Pinheiro FP, Rocha AG, Freitas RB. Meningitis associated with Oropouche virus infection. Revista do Instituto de Medicina Tropical de Sao Paulo. 1982;24(4):246-51. PubMed Central PMCID: PMC6818668.
67. Watts DM, Phillips I, Callahan JD, Griebenow W, Hyams KC, Hayes CG. Oropouche virus transmission in the Amazon River basin of Peru. 1997;1(2):148-52.
68. Anderson CR, Spence L, Downs WG, Aitkbn THG. Oropouche virus: a new human disease agent from Trinidad, West Indies. American Journal of Tropical Medicine and Hygiene. 1961;10(4):574-8 pp. doi: 10.4269/ajtmh.1961.10.574. PubMed PMID: CABI:19622701196.

69. Pinheiro FP, Travassos da Rosa APA, Gomes MLC, LeDuc JW, Hoch AL. Transmission of Oropouche virus from man to hamster by the midge Culicoides paraensis. Science, USA. 1982;215(4537):1251-3. doi: 10.1126/science.6800036. PubMed PMID: CABI:19822901977.
70. Cardoso BF, Serra OP, da Silva Heinen LB, Zuchi N, de Souza VC, Naveca FG, et al. Detection of Oropouche virus segment S in patients and in Culex quinquefasciatus in the state of Mato Grosso, Brazil. Memorias do Instituto Oswaldo Cruz. 2015;110(6):745-54. doi: 10.1590/0074-02760150123. PubMed PMID: BIOSIS:PREV201600059303.
71. Aguilar PV, Barrett AD, Saeed MF, Watts DM, Russell K, Guevara C, et al. Iquitos virus: A novel reassortant orthobunyavirus associated with human illness in Peru. PLoS Neglected Tropical Diseases. 2011;5(9). doi: 10.1371/journal.pntd.0001315. PubMed Central PMCID: PMC21949892.
72. Navarro JC, Giambalvo D, Hernandez R, Auguste AJ, Tesh RB, Weaver SC, et al. Isolation of Madre de Dios Virus (Orthobunyavirus; Bunyaviridae), an Oropouche Virus Species Reassortant, from a Monkey in Venezuela. American Journal of Tropical Medicine and Hygiene. 2016;95(2):328-38. doi: 10.4269/ajtmh.15-0679. PubMed PMID: WOS:000400206200014.
73. Tilston-Lunel NL, Hughes J, Acrani GO, da Silva DE, Azevedo RS, Rodrigues SG, et al. Genetic analysis of members of the species Oropouche virus and identification of a novel M segment sequence. J Gen Virol. 2015;96(Pt 7):1636-50. Epub 2015/03/05. doi: 10.1099/vir.0.000108. PubMed PMID: 25735305; PubMed Central PMCID: PMCPMC4635451.
74. Seymour C, Peralta PH, Montgomery GG. Viruses isolated form Panamanian sloths. American Journal of Tropical Medicine and Hygiene. 1983;32(6):1435-44. doi: 10.4269/ajtmh.1983.32.1435. PubMed Central PMCID: PMC6316795.
75. Bakonyi T, Kolodziejek J, Rudolf I, Bercic R, Nowotny N, Hubalek Z. Partial genetic characterization of sedlec virus (Orthobunyavirus, Bunyaviridae). Infection, Genetics and Evolution. 2013;19:244-9. doi: 10.1016/j.meegid.2013.07.025. PubMed PMID: CABI:20133415545.
76. Behar A, Leibovich BB, Edery N, Yanase T, Brenner J. First genomic detection of Peaton virus in a calf with hydranencephaly in Israel. Veterinary Medicine and Science. 2019;5(1):87-92. doi: 10.1002/vms3.129. PubMed Central PMCID: PMC30394673.
77. Yoshizawa N, Shinoto M, Katayama A, Bekku R, Inatani K. An abnormal birth in bovine suspected of being caused by Peaton virus first occurred in Shikoku region, Japan. Journal of Veterinary Medical Science. 2022;84(2):223-7. doi: 10.1292/jvms.21-0420.
78. Jun K, Yanaka T, Lee KK, Lee JB. Seroprevalence of bovine arboviruses belonging to genus orthobunyavirus in South Korea. Journal of Veterinary Medical Science. 2018;80(10):1619-23. doi: 10.1292/jvms.17-0542.
79. St George TD, Standfast HA, Cybinski DH, Filippich C, Carley JG. Peaton virus: A new simbu group arbovirus isolated from cattle and culicoides hrevitarsis in australia. Australian Journal of Biological Sciences. 1980;33(2):235-44. doi: 10.1071/BI9800235. PubMed Central PMCID: PMC7436869.
80. Nishi D, Egashira T. Serological survey of bovine ephemeral fever, Akabane, Aino, Chuzan, and Peaton viruses in swine in Saga prefecture. Journal of the Japan Veterinary Medical Association. 2011;64(7):540-4. PubMed PMID: CABI:20113256883.
81. Lee VH. Isolation of viruses from feld populations of Culicodes Diptera Ceratopogonidae in Nigeria. Journal of Medical Entomology. 1979;16(1):76-9. doi: 10.1093/jmedent/16.1.76. PubMed PMID: BIOSIS:PREV198069053977.
82. Rexhepi A, Wernike K, Berxholi K, Marku N, Spahiu J, Sherifi K. First evidence of Schmallenberg virus infection in domestic ruminants in Kosovo and Albania. Veterinaria Italiana. 2021;57(1):13-7. doi: 10.12834/VetIt.1346.7413.3.
83. Steinrigl A, Schiefer P, Schleicher C, Peinhopf W, Wodak E, Bago Z, et al. Rapid spread and association of Schmallenberg virus with ruminant abortions and foetal death in Austria in 2012/2013. Preventive Veterinary Medicine. 2014;116(4):350-9. doi: 10.1016/j.prevetmed.2014.03.006. PubMed PMID: WOS:000344443100004.

84. Conraths FJ, Beer M, Peters M. "Schmailenberg-virus": A novel infectious disease in ruminants. Tierarztliche Umschau. 2012;67(5):147-50.
85. Zhai SL, Lv DH, Wen XH, Zhu XL, Yang YQ, Chen QL, et al. Preliminary serological evidence for Schmallenberg virus infection in China. Tropical Animal Health and Production. 2018;50(2):449-53. doi: 10.1007/s11250-017-1433-2.
86. Bedekovic T, Lojkic I, Lemo N, Jemersic L, Keros T, Balatinec J, et al. Schmallenberg virus infection - case report in the Republic of Croatia

Infekcija Schmallenberg virusom - opis slucaja u Republici Hrvatskoj. Veterinarska Stanica. 2013;44(2):135-9. PubMed PMID: CABI:20133178237.
87. Chaintoutis SC, Kiossis E, Giadinis ND, Brozos CN, Sailleau C, Viarouge C, et al. Evidence of Schmallenberg virus circulation in ruminants in Greece. Tropical Animal Health and Production. 2014;46(1):251-5. doi: 10.1007/s11250-013-0449-5.
88. Al-Baroodi SY. Seroprevelance of Schmallenberg virus infection as emerging disease in cattle in Iraq. Iraqi Journal of Veterinary Sciences. 2021;35(3):495-9. doi: 10.33899/ijvs.2020.127071.1454.
89. Barrett D, More SJ, O'Neill R, Bradshaw B, Casey M, Keane M, et al. Prevalence and distribution of exposure to Schmallenberg virus in Irish cattle during October 2012 to November 2013. BMC Veterinary Research. 2015;11(1). doi: 10.1186/s12917-015-0564-9.
90. Molini U, Capobianco A, Dondona, Hilbert R, Monaco F. Antibodies against Schmallenberg virus detected in cattle in the Otjozondjupa region, Namibia. Journal of the South African Veterinary Association. 2018;89. doi: 10.4102/jsava.v89i0.1666.
91. Larska M, Jasik A, Zmudziński JF. Clinical cases of Schmallenberg virus infections in Poland. Med Weter. 2013;69(7):428-31.
92. Bouchemla F, Agoltsov VA, Larionov SV, Popova OM, Shvenk EV. Epizootiological study on spatiotemporal clusters of Schmallenberg virus and Lumpy skin diseases: The case of Russia. Vet World. 2018;11(9):1229-36. Epub 2018/11/10. doi: 10.14202/vetworld.2018.1229-1236. PubMed PMID: 30410226; PubMed Central PMCID: PMCPMC6200570.
93. Toplak I, Cociancich V, Rihtaric D, Tomislav P. First detection of Schmallenberg virus in Slovenia. Petrovic T, editor2013. 40-5 p.
94. Azkur AK, Albayrak H, Risvanli A, Pestil Z, Ozan E, Yılmaz O, et al. Antibodies to Schmallenberg virus in domestic livestock in Turkey. Tropical Animal Health and Production. 2013;45(8):1825-8. doi: 10.1007/s11250-013-0415-2.
95. Elbers ARW, Meiswinkel R, van Weezep E, van Oldruitenborgh-Oosterbaan MMS, Kooi EA. Schmallenberg virus in Culicoides spp. Biting midges, the Netherlands, 2011. Emerging Infectious Diseases. 2013;19(1):106-9. doi: 10.3201/eid1901.121054.
96. Herder V, Wohlsein P, Peters M, Hansmann F, Baumgaertner W. Salient Lesions in Domestic Ruminants Infected With the Emerging So-called Schmallenberg Virus in Germany. Veterinary Pathology. 2012;49(4):588-91. doi: 10.1177/0300985812447831. PubMed PMID: WOS:000305489100003.
97. Helmer C, Eibach R, Humann-Ziehank E, Tegtmeyer PC, Bürstel D, Mayer K, et al. Seroprevalence of Schmallenberg virus infection in sheep and goats flocks in Germany, 2012–2013. Veterinary Medicine and Science. 2016;2(1):10-22. doi: 10.1002/vms3.14.
98. Linden A, Desmecht D, Volpe R, Wirtgen M, Gregoire F, Pirson J, et al. Epizootic spread of Schmallenberg virus among wild cervids, Belgium, Fall 2011. Emerging Infectious Diseases. 2012;18(12):2006-8. doi: 10.3201/eid1812.121067.
99. Molenaar FM, La Rocca SA, Khatri M, Lopez J, Steinbach F, Dastjerdi A. Exposure of Asian elephants and other exotic ungulates to Schmallenberg virus. PLoS ONE. 2015;10(8). doi: 10.1371/journal.pone.0135532.
100. Dominguez M, Hendrikx P, Zientara S, Calavas D, JayM, Touratier A, et al. Preliminary estimate of Schmallenberg virus infection impact in sheep flocks – France. 2012;171(17):426-. doi: https://doi.org/10.1136/vr.100883.

101. Laloy E, Bréard E, Trapp S, Pozzi N, Riou M, Barc C, et al. Fetopathic effects of experimental Schmallenberg virus infection in pregnant goats. Veterinary Microbiology. 2017;211:141-9. doi: 10.1016/j.vetmic.2017.10.011.
102. Muskens J, Smolenaars AJG, Van Der Poel WHM, Mars MH, Van Wuijckhuise L, Holzhauer M, et al. Diarrhea and loss of production on Dutch dairy farms caused by the Schmallenberg virus. Tijdschrift voor Diergeneeskunde. 2012;137(2):112-5.
103. Dandawate CN, Rajagopalan PK, Pavri KM, Work TH. Virus isolations from mosquitoes collected in North Arcot district, Madras state, and Chittoor district, Andhra Pradesh between November 1955 and October 1957. Indian journal of medical research (New Delhi, India : 1994). 1969;57(8):1420-6.
104. Yanase T, Fukutomi T, Yoshida K, Kato T, Ohashi S, Yamakawa M, et al. The emergence in Japan of Sathuperi virus, a tropical Simbu serogroup virus of the genus Orthobunyavirus. Archives of Virology. 2004;149(5):1007-13. doi: 10.1007/s00705-003-0266-7. PubMed Central PMCID: PMC15098114.
105. Yanase T, Maeda K, Kato T, Nyuta S, Kamata H, Yamakawa M, et al. The resurgence of Shamonda virus, an African Simbu group virus of the genus Orthobunyavirus, in Japan. Archives of Virology. 2005;150(2):361-9. doi: 10.1007/s00705-004-0419-3. PubMed PMID: BIOSIS:PREV200500149266.
106. Hirashima Y, Kitahara S, Kato T, Shirafuji H, Tanaka S, Yanase T. Congenital malformations of calves infected with Shamonda virus, southern Japan. Emerging Infectious Diseases. 2017;23(6):993-6. doi: 10.3201/eid2306.161946.
107. Golender N, Bumbarov V, Assis I, Beer M, Khinich Y, Koren O, et al. Shuni virus in Israel: Neurological disease and fatalities in cattle. Transboundary and Emerging Diseases. 2019;66(3):1126-31. doi: 10.1111/tbed.13167.
108. van Eeden C, Williams JH, Gerdes TGH, van Wilpe E, Viljoen A, Swanepoel R, et al. Shuni virus as cause of neurologic disease in horses. Emerging Infectious Diseases. 2012;18(2):318-21. doi: 10.3201/eid1802.111403.
109. Moore DL, Causey OR, Carey DE, Reddy S, Cooke AR, Akinkugbe FM, et al. Arthropod-borne viral infections of man in Nigeria, 1964-1970. Ann Trop Med Parasitol. 1975;69(1):49-64. Epub 1975/03/01. doi: 10.1080/00034983.1975.11686983. PubMed PMID: 1124969.
110. Rodrigues FM, Singh PB, Dandawate CN, Soman RS, Bhatt PN. Kaikalur virus: a new arthropod borne virus belonging to the Simbu group isolated in India from Culex tritaeniorhynchus (Giles). Indian Journal of Medical Research. 1977;66(5):719-25. PubMed Central PMCID: PMC608738.
111. Weinbren MP, Heymann CS, Kokernot RH, Paterson HE. Studies on arthropod-borne viruses of Tongaland. VII. Simbu virus, a hitherto unknown agent isolated from Aedes (Banksinella) circumluteolus Theo. S African Jour Med Sci. 1957;22((2/3)):93-102. PubMed PMID: BIOSIS:PREV19593300030239.
112. Mitchell CJ, Monath TP, Sabattini MS, Cropp CB, Daffner JF, Calisher CH, et al. Arbovirus Investigations in Argentina, 1977-1980: II. Arthropod Collections and Virus Isolations from Argentine Mosquitoes. The American journal of tropical medicine and hygiene. 1985;34(5):945-55. doi: 10.4269/ajtmh.1985.34.945.
113. Carey DE, Reuben R, George S, Shope RE, Myers RM. Kammavanpettai, Kannamangalam, Sembalam and Thimiri viruses: four unrelated new agents isolated from birds in India. The Indian journal of medical research. 1971;59(11):1708-11. Epub 1971/11/01. PubMed PMID: 5143162.
114. Standfast HA, Dyce AL. Isolation of Thimiri virus from Culicoides histrio (Diptera: Ceratopogonidae) collected in Northern Australia. 1982;1(2):212.
115. Shope R, Andrade A, Bensabath G, editors. The serological response of animals to virus infection in Utinga forest, Belém, Brazil. Simposio sobre a biota Amazonica; 1966; Belém: Tropical Biology.
