# Supplementary material for: Revisiting the Importance of Orthobunyaviruses for Animal Health: A Scoping Review of Livestock Disease, Diagnostic Tests, and Surveillance Strategies for the Simbu Serogroup
Source: Viruses. 2024 Feb 15;16(2):294. doi: 10.3390/v16020294 (PMC10892073; doi:10.3390/v16020294)
Supplement: Supplementary file 1 [file viruses-16-00294-s001.zip › Supplementary Table S2.pdf]

Supplementary Table 2. Data extracted from eligible publications in a scoping review to identify veterinary-important Simbu group viruses. This information was used to produce a summary of the associated disease presentations, monitoring methods, and diagnostic assays of the SGVs with veterinary importance.

**Variable**	**Description**	**Unit/Levels**
Authors	The names of the authors listed on the publication	Free text list
Publication Date	The date for which the publication was published	Date/Month/Year format
Publication Year	The year for which the publication was published	Date/Month/Year format
Country	A list of the country affiliations of the authors	Free text list
Continent	The continent in which the study was conducted	Americas<br>Africa<br>Asia<br>Australia/Oceania<br>Europe
Title	The title of the publication	Free text list
Journal	The name of the journal of publication	Free text list
Virus name	The Simbu group virus/es investigated	see supplementary table 1 for the 33 viruses included in the serogroup
**Theme: The veterinary important SGVs and the associated animal disease presentation**		
Study type	Study type/design	Descriptive<br>Observational<br>Experimental<br>Theoretical
Infection type	Natural exposure or experimental challenge	Natural<br>Experimental
Case definition[1]	Diagnostic criterion applied to recruit cases into this review to provide a systematic description of disease presentations associated with SGV infection	Clinical presentation only<br>Antibody detection only<br>Agent detection only<br>Consistent clinical presentation with confirmation of viral infection with a SGV
Diagnostic test	Test type(s) utilized for confirmation of disease due to the virus of interest	Free text list
Start	The start date of the period of observation	Date/Month/Year format
End	The end date of the period of observation	Date/Month/Year format
Season	The season in which the observations of animal disease occurred.<br>The date of disease report was used to determine the season based on the period and location of the observation.	Summer<br>Autumn<br>Spring<br>Winter
Host species	Species of animal host with disease	Bovine<br>Ovine<br>Caprine<br>Porcine<br>Equine
Host age group[2]	Age group of the animal host with disease	Foetal: unborn<br>Juvenile: born to <12 months of age<br>Adult: 12 months of age

Disease system[2]	The organ system involved	Circulatory<br>Respiratory<br>Gastrointestinal<br>Musculoskeletal<br>Nervous<br>Urinary<br>Reproductive<br>Congenital<br>Non-specific
Clinical signs[2]	A list of all clinical signs reported	Free text list
Frequency of clinical signs listed	The count of the observations of clinical signs by the number of cases	Numeric
Differentials considered	What other diseases were considered	Free text list
Diagnostic tests	Assay types used to diagnose the virus of interest	Free text list
Histopathological description	The inclusion of histopathological description of disease	Yes<br>No
Economic losses estimated	The inclusion of an estimate or economic assessment of the impact of associated disease	Yes, with information collected in a free text list<br>No

[1] This assessment was completed to ensure that cases of clinical disease described was confirmed to be a result of infection with the virus of interest. The four diagnostic criteria are based on the unified case definitions proposed by European agencies during data collection for the Schmallenberg virus epidemic [21]. The diagnostic challenge in identifying a SGV as a cause of disease were exemplified by the different and varying case definitions used by affected countries during the outbreak in Europe. In January 2012, different countries had varying criteria for what constituted a confirmed case following Schmallenberg virus infection. These criteria depended on factors such as the age of the host (offspring or adult) or the location of the infection (within or outside the known range). Additionally, laboratory confirmation of the viral infection was achieved through a variety of agent and/or antibody tests.

[2] To provide a distinction between maternal and foetal outcomes of infection, clinical signs relating to reproductive and neurological disease were categorised given the context provided from the host age group. This allowed for congenital disease to be considered as a foetal outcome and reproductive disease to be measured as a maternal effect.

Theme: Diagnostic tests – development and validation studies

Test scenario	Reason for testing	Monitoring populations for infection/exposure<br>Diagnosis in animals where disease is suspected
Sample types	List of the samples utilized in test development or validation	Free text list
Test types	List of the test types investigated	Free text list
Test level	Test is designed to detect SGVs either at a group level or to differentiate a specific named virus	Group<br>Virus
Test target	Test designed for serology or agent detection	Serology<br>Agent
Test details	Details such as the test manufacturer, reagents, primer, and probe sequences	Free text list
Test validation	If the assay has undergone validation and if so, to which stage along the validation pathway as defined by WOAH guidelines [22]	None: No validation data provided<br>Stage 1: Analytical characteristics<br>Stage 2: Diagnostic characteristics and cut-off values<br>Stage 3: Reproducibility<br>Stage 4: Implementation

Theme: Monitoring methods

Virus	Simbu group virus of interest for the activity	Free text list
Survey Area	Area of interest for the study	Local region (intra-country)<br>Country<br>Multinational

		Continent
Survey location	Details of the country and region where the activity occurred	Free text list
Survey period	Length of time over the course of the study	Numeric (months)
Sample collection	The type sample collection used in the study:<br>Active if the samples were primarily collected for the surveillance activity.<br>Passive if the samples were collected for another purpose (diagnostic investigation or a non-SGV surveillance program)	Active<br>Passive
Survey test	Test type(s) utilized in the monitoring activity	Free text list
Animal	Animal species from where samples were collected from	Free text list
Sample frequency	Frequency of sampling used for the study	Single<br>Serial – repeated/ daily/ weekly/ monthly/ quarterly/ Yearly<br>Paired
Sample size calculation	If sample size calculations were considered and the details of the:<br>- Anticipated prevalence<br>- Allowable error<br>- Level of confidence	Yes/No<br><br>Numeric for the information on the sample size calculation
Sample size	Sample size	Numeric
Seroprevalence[3]	Reported seroprevalence in each study was charted by:<br>- Country<br>- Animal species<br>- Simbu group virus	Numeric

[3] A quantitative summary of seroprevalence by country, animal species, and Simbu group virus was prepared using R with data extracted (Supplementary Figure 1).
