# Supplementary material for: Revisiting the Importance of Orthobunyaviruses for Animal Health: A Scoping Review of Livestock Disease, Diagnostic Tests, and Surveillance Strategies for the Simbu Serogroup
Source: Viruses. 2024 Feb 15;16(2):294. doi: 10.3390/v16020294 (PMC10892073; doi:10.3390/v16020294)
Supplement: Supplementary file 1 [file viruses-16-00294-s001.zip › Supplementary Table S3.pdf]

Named virus	Challenge type (route)	Host Species	Case presentation and signalment	Microscopic descriptions			Ref.
				Brain	Spinal cord	Skeletal muscle	
Peaton virus	Natural<br>(in-utero)	Bovine	Aborted, malformed calf Japanese Black bull × Holstein cow.<br>Gestational age 285 days	No description provided	No description provided	Loss of skeletal muscle fibres accompanied by adipose replacement	[1]
Shuni virus	Experimental<br>(subcutaneous)	Bovine	7-11 months old calves	Mild to moderate, nonsuppurative meningoencephalitis.<br>- Perivascular cuffs<br>- Glial nodule formation	Mild inflammatory changes	No description provided	[2]
Shuni virus	Natural	Bovine	5-month-old male Holstein-Friesian calf	Acute-subacute meningoencephalitis<br>- Perivascular cuffs<br>- Neutrophils, with few lymphocytes, infiltration of the cerebellar meninges<br>- Aggregates of lymphocytes and neutrophils in brain stem accompanied by white matter oedema	No description provided	No description provided	[3]
Shuni virus	Natural	Bovine	16-month-old Holstein-Frisian heifer	Encephalitis<br>- Thick perivascular cuffs (macrophages and neutrophils)<br>- Cerebral and meningeal haemorrhages<br>- Diffuse, mild gliosis and rare micro-abscesses accompanied by white matter oedema	No description provided	No description provided	[3]
Schmallenberg virus	Experimental<br>(subcutaneous)	Ovine	4 months old Charollais sheep	No lesions were observed	No lesions were observed	No lesions were observed	[4]
Schmallenberg virus	Experimental<br>(subcutaneous)	Bovine	6 month old Holstein calves	Non-specific lesions<br>- Focal gliosis in the brain stem with another region of hypercellularity<br>- Cerebral perivascular cuffing with mononuclear cells with no evidence of vasculitis	No lesions were observed	No lesions were observed	[4]
Schmallenberg virus	Experimental<br>(in-utero, day 28 and day 42 of pregnancy)	Caprine	Stillborn animals Alpine/Saanen	Porencephaly associated with multiple foci of haemorrhage.	No lesions were observed	No lesions were observed	[5]
Shamonda virus	Natural<br>(in-utero)	Bovine	Aborted, malformed foetuses and stillborn animals - delivered at or beyond term.	Lesions in the cerebrum and brain stem<br>- Calcification of nerve cells<br>- Perivascular infiltration of mononuclear cells<br>- Gliosis	Severe degeneration in the ventral horn	Fatty replacement, atrophy, myositis	[6]

			Gestation age: 275–299 days				
Schmallenberg virus	Natural (in-utero)	Bovine	Aborted, malformed foetuses and stillborn animals	- cerebellar and cerebral hypoplasia,<br>- meningeal fibrosis,<br>- porencephaly<br>- micromyelia and lack of neurons<br>- demyelination without inflammation.	Micromyelia, lack of neurons in the dorsal horns	- Myofibrillar hypoplasia<br>- Loss of skeletal muscle fibres replaced by myxoid tissue	[7]
Schmallenberg virus	Natural (in-utero)	Bovine	Aborted and malformed foetus (female calf, Charolais hybrid)	- Cerebrum: moderate microgliosis<br>- Cerebellum:<br>o Reduced thickness to the molecular layer<br>o Loss of Purkinje cells	- Cervical: devoid of grey and white matter<br>- Thoracic: lateral symmetrical demyelination (reduced grey matter)<br>- Lumbar: inadequate development of grey and white matter	- Severe myofibrillar hypoplasia<br>- Loss of skeletal muscle fibres replaced with lipocytes and connective tissue<br>- Dystrophic mineralisation of myocytes	[8]
Schmallenberg virus	Natural (in-utero)	Bovine	Aborted, malformed foetuses and stillborn animals	Limited to gross features:<br>- Porencephaly<br>- Hydranencephaly<br>- Hydrocephaly	Decreased neurons	Increased fibre size variation with connective tissue and adipocyte infiltration	[9]
Akabane virus	Natural	Cattle	4 months to 6 year-old animals	Moderate-to-severe non-suppurative meningoencephalomyelitis<br>- Perivascular infiltration of lymphocytes and histiocytes with fewer plasma cells<br>- Multifocal gliosis<br>- Neuronal necrosis<br>- Neuronophagia	- Lymphohistiocytic perivascular cuffing,<br>- Gliosis<br>- Neuronal generation	No description provided	[10]
Schmallenberg virus	Natural (in-utero)	Bovine	Aborted, malformed foetuses and stillborn animals	- Mild, diffuse, lymphohistiocytic meningitis without perivascular or parenchymal infiltration of the brain<br>- Neuronal necrosis and chromatolysis<br>- mild to severe, diffuse astrogliosis and/or microgliosis	Micromyelia, bilateral reduction of grey matter and white matter (reduced neurons in the ventral horns)	Severe reduction of myofibrils with diminished myofiber diameter	[11]
Schmallenberg virus	Natural (in-utero)	Ovine	Aborted, malformed foetuses and stillborn animals	- Perivascular infiltration of the grey and white matter (lymphohistiocytic)<br>- Perivascular cuffs<br>- Glial nodules<br>- Neuronal necrosis and chromatolysis<br>- mild to severe, diffuse astrogliosis and/or microgliosis	No description provided	Severe reduction of myofibrils with diminished myofiber diameter	[11]
Schmallenberg virus	Natural (in-utero)	Caprine	Aborted, malformed foetuses and stillborn animals	- mild to severe, diffuse astrogliosis and/or microgliosis	No description provided	No description provided	[11]

Akabane virus	Natural	Bovine	55 days to 15-month-old calves. (Japanese Black, Holstein, mixed breed)	Non-suppurative encephalitis<br>- Perivascular cuffing (lymphocytes and macrophages)<br>- Diffuse gliosis<br>- degeneration and/or necrosis of neurons<br>- vacuolation of the neuropil	Ventral horn - neuronal necrosis and neuronophagia	Mild degeneration of skeletal muscle fibres	[12]
Akabane virus	Natural	Bovine	3 days to 4-month-old calves	Moderate to severe lymphohistiocytic encephalomyelitis<br>- perivascular infiltration (mixed lymphocytes and histiocytes with fewer plasma cells)<br>- Multifocal gliosis<br>- Neuronal degeneration<br>- Occasional neuronophagia	Lymphohistiocytic perivascular cuffing, moderate gliosis and neuronal necrosis and loss were also observed in the spinal cord, mostly in the ventral horn grey matter	Mild inflammatory reaction associated with muscle atrophy of limb muscle	[13]
Akabane virus	Natural	Bovine	2–7-year-old adult cows	- moderate to severe perivascular infiltration of primarily lymphocytes and histiocytes with fewer plasma cells,<br>- Mild perivascular lymphohistiocytic cuffing<br>- Multifocal gliosis,<br>- Neuronal necrosis,<br>- Occasional neuronophagia with microglial cells and neutrophils	- lymphohistiocytic perivascular cuffing,<br>- gliosis,<br>- neuronal necrosis and loss<br>Inflammatory changes were more severe in the spinal cord than in the brain	Severely atrophic, with mucinous change in the stroma	[14]
Akabane virus	Natural (in-utero)	Bovine	Stillborn animals	- perivascular cuffs of lymphocytes, plasma cells and macrophages<br>- diffuse and focal proliferation of glial cells<br>- pyknosis of neuronal cells<br>- vacuolation of neuropils	Bilaterally in the ventral horn:<br>- perivascular cuffing<br>- diffuse and focal proliferation of glial cells<br>- pyknosis of neuronal cells<br>- decreased number of neurons	No lesions were observed	[15]
Akabane virus	Natural	Bovine	5-month-old bull calf	Severe lymphohistiocytic meningoencephalomyelitis<br>- perivascular infiltration of mononuclear cells<br>- diffuse to multifocal gliosis<br>- neuronal necrosis	Inflammatory changes in all regions:<br>- marked gliosis,<br>- perivascular cuffing by mononuclear cells,<br>Neuronal necrosis in the bilateral ventral horns	No lesions were observed	[16]
Akabane virus	Natural	Bovine	One day old calves	Severe lymphohistiocytic meningoencephalomyelitis<br>- perivascular infiltration of mononuclear cells<br>- diffuse to multifocal gliosis<br>- neuronal necrosis	Ventral horn grey matter:<br>- diffuse gliosis<br>- perivascular cuffing with mononuclear cells<br>- neuronal cell loss	- Severe diffuse atrophic or dysplastic muscles<br>- myofibrillar hypoplasia<br>- mucinous change in the stromal connective tissues.<br>- Mild infiltration of small number of histiocytes and	[16]

						neutrophils was found occasionally around blood vessels	
Aino virus	Natural (in-utero)	Bovine	Aborted calf Gestational age 187 days.	- Necrotic foci with macrophages (gitter cells). The necrotic foci showed some calcification of neuronal cells<br>- Multifocal lymphatic perivascular cuffing in the brain and meninges	No lesions were observed	No lesions were observed	[17]
Akabane virus	Natural (in-utero)	Bovine	4-12 month-old dairy calves	- Non-suppurative encephalitis<br>- Moderate perivascular infiltration	- Neuronal degeneration<br>- Neuronophages	- muscle myofibers were discontinued or broken.<br>- mild proliferation of satellite cells<br>- myofiber degeneration and atrophy	[18]
Akabane virus	Experimental (intracranial)	Bovine	A 2-day-old, Holstein calf and a 6-month-old, Holstein calf	- Severe perivascular infiltration (lymphocytes)<br>- Glial nodules in the grey matter	Lesions more severe in the ventral horn than the dorsal horn<br>- Glial nodules<br>- Neuronal degeneration	No description provided	[19]
Akabane virus	Experimental (in-utero, days 32-33 of pregnancy)	Ovine	Sacrificed foetuses	Day 9: Focal destruction of the brain cells<br><br>Day 21: Generalised destruction of the brain cells, with<br>- Necrosis<br>- Mineralisation<br>- Oedema<br>- General disruption of the structure	No description provided	Day 21: necrosis, increased cellularity and disruption of cell structure	[20]
Akabane virus	Natural (In-utero, 0-280 days of pregnancy)	Bovine	Malformed foetuses	Diffuse, suppurative meningoencephalitis, with<br>- Necrosis<br>- Suppuration and phagocytosis in the meninges and ventricular system	Mild depletion of ventral horn neurones, axons and myelin	No description provided	[21]
Akabane virus	Natural (in-utero)	Ovine	Malformed foetuses	No description provided	Neuronal loss in the ventral horn	Severe skeletal muscle neurogenic atrophy and replacement by adipose tissue	[22]
Akabane virus	Natural (in-utero)	Bovine	Malformed foetuses	- Perivascular cuffing with histiocytes, plasma cells and lymphocytes<br>- Gliosis<br>- Nerve cell degeneration – chromatolysis<br>- Necrotic foci with infiltration of lipid-laden macrophages<br>- Cystic cavities	Neurons were absent or sparse in the ventral horn	Dysplastic changes – tentatively termed:<br>- wuishokinsho (runt-muscle disease)<br>- wuishokinseni (runt-muscle fiber)	[23]
Akabane virus	Experimental (in-utero, days 32-36 of pregnancy)	Ovine	Sacrificed foetuses	- hypoplasia and degeneration<br>- perivascular cuffing<br>- cysts and malacia<br>- gliosis	- Neurons were absent in the ventral horn<br>- Myelination deficiency<br>- Perivascular cuffing	Muscle neuronal atrophy and degeneration	[24]

					- Gliosis<br>- Oedema<br>- Neuronal necrosis<br>- Neuronophagia		
Aino virus	Natural<br>(in-utero)	Bovine	Malformed newborn calves with neurologic dysfunction	Mild non-suppurative encephalitis<br>- lack of folial development of the cerebellum<br>- Decreased width of the molecular layer<br>- loss of Purkinje cells<br>- loss of cells in the granular layer	No description provided	No description provided	[25]
Akabane	Experimental<br>(in-utero, days 30-36 of pregnancy)	Ovine	Sacrificed newborn lambs	Meninges – fused membranes with focal areas of necrotic debris and fibrous thickenings	Reduced size, marked reduction of myelination of the ventral cord and neurons were reduced in the ventral horn	Muscle bundles varied in size, and areas of atrophy and degeneration were present.<br>There was a loose arrangement of mixed tissues, consisting of some adipose cells and fibrous tissues among the muscle cells.	[26]
Aino virus	Experimental<br>(intracerebral)	Bovine	2-6 month old Japanese Brown X Holstein calves	- Swelling of the vascular endothelial cells<br>- Cell infiltration (histiocytes, lymphocytes, large mononuclear cells and plasma cells)<br>- Proliferation of neuroglia to form nodules.<br>- Degeneration of nerve cells<br>- Mild haemorrhage dispersedly in parts	No description provided	No lesions were observed	[27]
Akabane virus	Experimental<br>(intracerebral)	Bovine	2-6 month old Japanese Brown X Holstein calves	- Congested blood capillaries with swollen endothelial cells<br>- Perivascular cuffing with large mononuclear cells, histiocytes, and lymphocytes<br>- Proliferation of neuroglia to form aggregations of cells	No description provided	No lesions were observed	[27]
Akabane virus	Natural<br>(in-utero)	Bovine	Malformed newborn calves with neurologic dysfunction	No significant lesions, but in several brains:<br>- small focal cavitations<br>- pigmented, iron-containing macrophages,<br>- mild to moderate perivascular cuffing	Smaller than normal with complete absence of all elements except the dorsal columns and small parts of dorsal horns.	Mild to moderate neurogenic skeletal muscle atrophy and/or adipose tissue replacement	[28]
Akabane virus	Natural<br>(in-utero)	Bovine	Sacrificed foetuses Gestational age: 92 days	- Encephalomyelitis<br>- Perivascular cuffing<br>- Gliosis		- Polymyositis – cell infiltration and degeneration	[29]

References:

1. Yoshizawa N, Shinoto M, Katayama A, Bekku R, Inatani K. An abnormal birth in bovine suspected of being caused by Peaton virus first occurred in Shikoku region, Japan. Journal of Veterinary Medical Science. 2022;84(2):223-7. doi: 10.1292/jvms.21-0420.
2. Sick F, Breithaupt A, Golender N, Bumbarov V, Beer M, Wernike K. Shuni virus-induced meningoencephalitis after experimental infection of cattle. Transboundary and Emerging Diseases. 2021;68(3):1531-40. doi: 10.1111/tbed.13823. PubMed Central PMCID: PMC32910551.
3. Golender N, Bumbarov V, Assis I, Beer M, Khinich Y, Koren O, et al. Shuni virus in Israel: Neurological disease and fatalities in cattle. Transboundary and Emerging Diseases. 2019;66(3):1126-31. doi: 10.1111/tbed.13167.
4. Endalew AD, Morozov I, Davis AS, Gaudreault NN, Wernike K, Bawa B, et al. Virological and serological responses of sheep and cattle to experimental Schmallenberg virus infection. Vector-Borne and Zoonotic Diseases. 2018;18(12):697-703. doi: 10.1089/vbz.2018.2297.
5. Laloy E, Bréard E, Trapp S, Pozzi N, Riou M, Barc C, et al. Fetopathic effects of experimental Schmallenberg virus infection in pregnant goats. Veterinary Microbiology. 2017;211:141-9. doi: 10.1016/j.vetmic.2017.10.011.
6. Hirashima Y, Kitahara S, Kato T, Shirafuji H, Tanaka S, Yanase T. Congenital malformations of calves infected with Shamonda virus, southern Japan. Emerging Infectious Diseases. 2017;23(6):993-6. doi: 10.3201/eid2306.161946.
7. Balseiro A, Royo LJ, Gómez Antona A, García Marín JF. First Confirmation of Schmallenberg Virus in Cattle in Spain: Tissue Distribution and Pathology. Transboundary and Emerging Diseases. 2015;62(5):e62-e5. doi: 10.1111/tbed.12185.
8. Wisloff H, Nordvik BS, Sviland S, Tonnessen R. First documented clinical case of Schmallenberg virus in Norway: fetal malformations in a calf. Veterinary Record. 2014;174(5):120-+. doi: 10.1136/vr.102149. PubMed PMID: WOS:000330573100021.
9. Bayrou C, Garigliany M-M, Sarlet M, Sartelet A, Cassart D, Desmecht D. Natural Intrauterine Infection with Schmallenberg Virus in Malformed Newborn Calves. Emerging Infectious Diseases. 2014;20(8):1327-30. doi: 10.3201/eid2008.121890. PubMed PMID: WOS:000339864000009.
10. Oem JK, Lee KH, Kim HR, Bae YC, Chung JY, Lee OS, et al. Bovine Epizootic Encephalomyelitis caused by Akabane Virus Infection in Korea. Journal of Comparative Pathology. 2012;147(2-3):101-5. doi: 10.1016/j.jcpa.2012.01.013.
11. Herder V, Wohlsein P, Peters M, Hansmann F, Baumgaertner W. Salient Lesions in Domestic Ruminants Infected With the Emerging So-called Schmallenberg Virus in Germany. Veterinary Pathology. 2012;49(4):588-91. doi: 10.1177/0300985812447831. PubMed PMID: WOS:000305489100003.
12. Kamata H, Inai K, Maeda K, Nishimura T, Arita S, Tsuda T, et al. Encephalomyelitis of Cattle Caused by Akabane Virus in Southern Japan in 2006. Journal of Comparative Pathology. 2009;140(2-3):187-93. doi: 10.1016/j.jcpa.2008.12.001.
13. Lee JK, Kim JH, Park BK, Yoo HS, Lee BC, Kim DY. Akabane viral encephalitis in calves in South Korea. Veterinary Record. 2007;161(7):236-8. doi: 10.1136/vr.161.7.236.
14. Lee JK, Park JS, Choi JH, Park BK, Lee BC, Hwang WS, et al. Encephalomyelitis Associated with Akabane Virus Infection in Adult Cows. Veterinary Pathology. 2002;39(2):269-73. doi: 10.1354/vp.39-2-269.
15. Noda Y, Yokoyama H, Katsuki T, Kurashige S, Uchinuno Y, Narita M. Demonstration of Akabane virus antigen using immunohistochemistry in naturally infected newborn calves. Veterinary Pathology. 2001;38(2):216-8. doi: 10.1354/vp.38-2-216.
16. Uchida K, Murakami T, Sueyoshi M, Tsuda T, Inai K, Acorda JA, et al. Detection of Akabane viral antigens in spontaneous lymphohistiocytic encephalomyelitis in cattle. Journal of Veterinary Diagnostic Investigation. 2000;12(6):518-24. doi: 10.1177/104063870001200605.

17. Noda Y, Uchinuno Y, Shirakawa H, Nagasue S, Nagano N, Ohe R, et al. Aino virus antigen in brain lesions of a naturally aborted bovine fetus. Veterinary Pathology. 1998;35(5):409-11. doi: 10.1177/030098589803500511.
18. Liao YK, Lu YS, Goto Y, Inaba Y. The isolation of Akabane virus (Iriki strain) from calves in Taiwan. Journal of Basic Microbiology. 1996;36(1):33-9. doi: 10.1002/jobm.3620360108.
19. Miyazato S, Miura Y, Hase M, Kubo M, Goto Y, Kono Y. Encephalitis of cattle caused by Iriki isolate, a new strain belonging to Akabane virus. Nippon juigaku zasshi The Japanese journal of veterinary science. 1989;51(1):128-36. doi: 10.1292/jvms1939.51.128. PubMed Central PMCID: PMC2494374.
20. Parsonson IM, McPhee DA, Della-Porta AJ, McClure S, McCullagh P. Transmission of Akabane virus from the ewe to the early fetus (32 to 53 days). Journal of Comparative Pathology. 1988;99(2):215-27. doi: 10.1016/0021-9975(88)90073-4.
21. Kirkland PD, Barry RD, Harper PA, Zelski RZ. The development of Akabane virus-induced congenital abnormalities in cattle. The Veterinary record. 1988;122(24):582-6. doi: 10.1136/vr.122.24.582. PubMed Central PMCID: PMC3137718.
22. Haughey KG, Hartley WJ, Della-Porta AJ, Murray MD. Akabane disease in sheep. Australian veterinary journal. 1988;65(5):136-40. doi: 10.1111/j.1751-0813.1988.tb14440.x. PubMed Central PMCID: PMC3135792.
23. Konno S, Moriwaki M, Nakagawa M. Akabane Disease in Cattle: Congenital Abnormalities caused by Viral Infection. Spontaneous Disease. Veterinary Pathology. 1982;19(3):246-66. doi: 10.1177/030098588201900304.
24. Parsonson IM, Della-Porta AJ, Snowdon WA. Akabane virus infection in the pregnant ewe. 2. Pathology of the foetus. Veterinary Microbiology. 1981;6(3):209-24. doi: 10.1016/0378-1135(81)90014-6.
25. Coverdale OR, Cybinski DH, St George TD. Congenital abnormalites in calves associated with Akabane virus and Aino virus. Australian Veterinary Journal. 1978;54(3):151-2. doi: 10.1111/j.1751-0813.1978.tb05538.x. PubMed PMID: WOS:A1978ES96400017.
26. Parsonson IM, Della-Porta AJ, Snowdon WA. Congenital abnormalities in newborn lambs after infection of pregnant sheep with Akabane virus. Infection and Immunity. 1977;15(1):254-62. doi: 10.1128/iai.15.1.254-262.1977.
27. Moriwaki M, Miura Y, Hayashi S, Ishitani R. Histopathological findings of calves infected experimentally with Aino virus. National Institute of Animal Health quarterly. 1977;17(3):95-106. PubMed Central PMCID: PMC917129.
28. Hartley WJ, Desaram WG, Della-Porta AJ, Snowdon WA, Shepherd NC. Pathology of congenital bovine episootic arthrogryposis and hydranenecphaly and its relationship to Akabane virus. Australian Veterinary Journal. 1977;53(7):319-25. doi: 10.1111/j.1751-0813.1977.tb00240.x. PubMed PMID: WOS:A1977DQ22200005.
29. Kurogi H, Inaba Y, Takahashi E, Sato K, Omori T, Miura Y, et al. Epizootic congenital arthrogryposis-hydranencephaly syndrome in cattle: Isolation of Akabane virus from affected fetuses. Archives of Virology. 1976;51(1-2):67-74. doi: 10.1007/BF01317835.
