# Supplementary material for: Revisiting the Importance of Orthobunyaviruses for Animal Health: A Scoping Review of Livestock Disease, Diagnostic Tests, and Surveillance Strategies for the Simbu Serogroup
Source: Viruses. 2024 Feb 15;16(2):294. doi: 10.3390/v16020294 (PMC10892073; doi:10.3390/v16020294)
Supplement: Supplementary file 1 [file viruses-16-00294-s001.zip › Supplementary Table S4.pdf]

Named virus	PCR		Forward primer 5' -3'		Reverse primer 5' -3'		Probe 5' -3'		Reference
Aino virus	RT	S	AISF132	CCCAACTCAATTTCGATACC	AISR780	TTTGGAACACCATACTGGGG			[1]
Aino virus	nested	S	AISF313	CCATCGTCTCTCAGGATATC	AISR657	ACAGCATTGAAGGCTGCACG			[1]
Aino virus	q-RT		CommonF	TGACTGCAGAAGARTGGATGA	CommonR	GAATCCA GATTTGGCCCA	AINV Probe	Cy5-ATGCGTCCGTGCA-BHQ2	[2]
Aino virus	q-RT		AI295F	GACACTGCCCTCACTCTCCAT	AI361R	TTTACATTGGTCTGCAACCCATT	AI317-FAM	CGTCTCTCAGGATATCTAGCAA	[3]
Aino virus	**T7**		AI179T	**TTAATACGACTCACTATAGGGAG**AAAGGCCAAGATGGTACTCAG	AI651L	GCTTAGCTGCCCAACAGCAT			[3]
Akabane virus		S	AKSF19	TAACTACGCATTGCAATGGC	AKSR740	TAAGCTTAGATCTGGATACC			[1]
Akabane virus	nested	S	AKSF177	GAAGGCCAAGATGGTCTTAC	AKSR407	GGCATCACAATTGTGGCAGC			[1]
Akabane virus	q-RT		CommonF	TGACTGCAGAAGARTGGATGA	CommonR	GAATCCAGATTTGGCCCA'	AKAV Probe	HEX-TCTAAGTTGGACGCCA-BHQ1	[2]
Akabane virus		S	AKAI206F	CACAACCAAGTGTCGATCTTA	AKAI560R	AAGTTGACATCCATTCCATC			[4]
Akabane virus		S	Akabane_AB289319F	GAACTCCACTATTAACTACGCATTG	Akabane_AB289319R	AAAGGTGTGCACCACATAGA			[5]
Akabane virus		S	Simbu1F	TCAACCAGAAGAAGGCCAAGAT	Simbu1R	GGGAAAATGGTTATTAACCACTGTAAA	AKAV S2 Probe	TTACATAAGACGCCACAACCA	[6]

Named virus	PCR		Forward primer 5’ -3’		Reverse primer 5’ -3’		Probe 5’ -3’		Reference
Akabane virus	q-RT		AK151F	GCTAGAGTCTTCTTCCTCAACCAGAA	AK231R	AAAAGTAAGATCGACACTTGGTTGTG	AK182VIC	CCAAGATGGTCTTACATAAG	[3]
Akabane virus	**T7**		AK100T	**TTAATACGACTCACTATAGGGAG**AGGGTATGTGGCRTTTATCAG	AK619L	TTGACTGCGTCCAACTTAGA			[3]
Ingwavuma virus		S	KF697141 F	AGTAGTGTACTCCACWATTCAA	KF697141R	GTAGTGTGCTCCCAATTCA			[5]
Pan-Simbu group		L	panOBV-L-2959 F	TTGGAGARTATGARGCTAARATGTG	panOBV-L-3274R	5’-TGAGCACTCCATTTNGACATRTC-3’			[7-9]
Pan-Simbu group	q-RT	S	Uni-S-59F	GATGTWCCWCAACGGAAT	Uni-S-254R	TGGGGAAAATGGTTATTAAC	Uni-S-probe	FAM-GAGTCTTCTTCCTCAA-MGB-NFQ	[10]
Pan-Simbu group		S	Simbu1 F	TCAACCAGAAGAAGGCCAAGAT	Simbu1 R	GGGAAAATGGTTATTAACCACTGTAAA	Simbu1 Probe	TAAGACGGCACAACCAAGTGT	[6]
Peaton virus		M	PEAV6F	GTCGACGAGTATTGCTTATCATC	PEAV6R-2	CATTGTGGACAACTTTAGTTTGGC	PEAV6 Probe	AATTTAAAGTCTTGTACTTGGC	[6]
Sabo virus		S	AF362396 F	GTGTACTCCACTATTAACTACGTACC	AF362396R	GAATTGGCGTGTCTCACATAGA			[5]
Sathuperi virus		S	HE795104 F	CACTACTGAAATATGTCAAGCCAATTC	HE795104R	CTCAACAGAAGCCTTGCAGTAT			[5]
Schmallenberg virus		S	Common F	TGACTGCAGAAGARTGGATGA	Common R	GAATCCA GATTTGGCCCA	SBV Probe	FAM-ACAGAAATAAAAGCTGCT-BHQ1-3’	[2]
Schmallenberg virus		M	SBV-M1-213 F	TCA ATT CAG CAA GTA ACA TAC AAT GG	SBV-M1-350R	CGT GGT CTG TCT TGG TTG ATG	SBV-M1-240FAM	FAM-AAG CAC TGG CCC GAA GTT TCA CCT-BHQ1	[7]

Named virus	PCR		Forward primer 5’ -3’		Reverse primer 5’ -3’		Probe 5’ -3’		Reference
Schmallenberg virus		L	SBV-L1-11F	TTG CCG TTT GAT TTT GAA GTT GTG	SBV-L1-155R	TCA GGG ATC GCA AAT TAA AGA ACC	SBV-L1-36FAM	FAM-TCA TCC GTG CTG ACC CTC TGC GAG-BHQ1	[7, 11, 12]
Schmallenberg virus		L	SBV-L1.2F	TCA GAA TTG CCG TTT GAT TTT GAA G	SBV-L1.4R	GTT GAG CGG CCC AAA TAT TTC C	SBV-L1-36FAM	FAM-TCA TCC GTG CTG ACC CTC TGC GAG-BHQ1	[7]
Schmallenberg virus		S	SBV-S-382F	TCA GAT TGT CAT GCC CCT TGC	SBV-S-469R	TTC GGC CCC AGG TGC AAA TC	SBV-S-408FAM	FAM-TTA AGG GAT GCA CCT GGG CCG ATG GT-BHQ1	[7, 9, 11-14]
Shamonda virus		S	NC018464F	CCACTATTAACTACAGAAATATGTCAAGCC	NC018464R	GGACCCGAAAGATGGTGAACTA			[5]
Shuni virus		S	KU937313F	AGTGTAYTCCACTATAGAACAAAGC	KU937313R	AGTGTGCTCCACATAGAACAAT			[5]
Simbu virus		S	NC018477F	AATGGCAAACCAATTCA	NC018477R	GGCGTACAACACATAGA			[5]

1. Akashi, H., et al., *Detection and differentiation of Aino and Akabane Simbu serogroup bunyaviruses by nested polymerase chain reaction.* Archives of Virology, 1999. **144**(11): p. 2101-2109.
2. Lee, J.H., et al., *Detection and differentiation of Schmallenberg, Akabane and Aino viruses by one-step multiplex reverse-transcriptase quantitative PCR assay.* BMC Veterinary Research, 2015. **11**(1).
3. Stram, Y., et al., *Detection and quantitation of Akabane and Aino viruses by multiplex real-time reverse-transcriptase PCR.* Journal of Virological Methods, 2004. **116**(2): p. 147-154.
4. Ohashi, S., et al., *Simultaneous detection of bovine arboviruses using single-tube multiplex reverse transcription-polymerase chain reaction.* Journal of Virological Methods, 2004. **120**(1): p. 79-85.
5. Camarão, A.A.R., et al., *Development and analytical validation of a group-specific RT-qPCR assay for the detection of the Simbu serogroup orthobunyaviruses.* Journal of Virological Methods, 2019. **271**.
6. Shirafuji, H., et al., *Broad-range detection of arboviruses belonging to Simbu serogroup lineage 1 and specific detection of Akabane, Aino and Peaton viruses by newly developed multiple TaqMan assays.* Journal of Virological Methods, 2015. **225**: p. 9-15.
7. Fischer, M., et al., *Development of a pan-Simbu real-time reverse transcriptase PCR for the detection of Simbu serogroup viruses and comparison with SBV diagnostic PCR systems.* Virology Journal, 2013. **10**.

8. Gurau, M.R., et al., *Studies concerning the optimisation of reverse transcription polymerase chain reaction technique of pan-Simbu virus group.* Scientific Bulletin. Series F. Biotechnologies, 2016. **20**: p. 329-332.
9. Wernike, K. and M. Beer, *International proficiency trial demonstrates reliable Schmallenberg virus infection diagnosis in endemic and non-affected countries.* PLoS ONE, 2019. **14**(6).
10. Golender, N., et al., *Development and validation of a universal S-segment-based real-time RT-PCR assay for the detection of Simbu serogroup viruses.* Journal of Virological Methods, 2018. **261**: p. 80-85.
11. De Regge, N., et al., *Schmallenberg virus circulation in Culicoides in Belgium in 2012: Field validation of a real time RT-PCR approach to assess virus replication and dissemination in midges.* PLoS ONE, 2014. **9**(1).
12. De Regge, N., et al., *Diagnosis of Schmallenberg virus infection in malformed lambs and calves and first indications for virus clearance in the fetus.* Veterinary Microbiology, 2013. **162**(2-4): p. 595-600.
13. Perera, C.L., et al., *Adaptation of a real-time RT-PCR assay for the detection of Schmallenberg virus.* Revista de Salud Animal, 2013. **35**(3): p. 201-205.
14. Aebischer, A., et al., *Rapid genome detection of Schmallenberg virus and bovine viral diarrhea virus by use of isothermal amplification methods and high-speed real-time reverse transcriptase PCR.* Journal of Clinical Microbiology, 2014. **52**(6): p. 1883-1892.
