# Supplementary figures and images for: Revisiting the Importance of Orthobunyaviruses for Animal Health: A Scoping Review of Livestock Disease, Diagnostic Tests, and Surveillance Strategies for the Simbu Serogroup
Source: Viruses. 2024 Feb 15;16(2):294. doi: 10.3390/v16020294 (PMC10892073; doi:10.3390/v16020294)

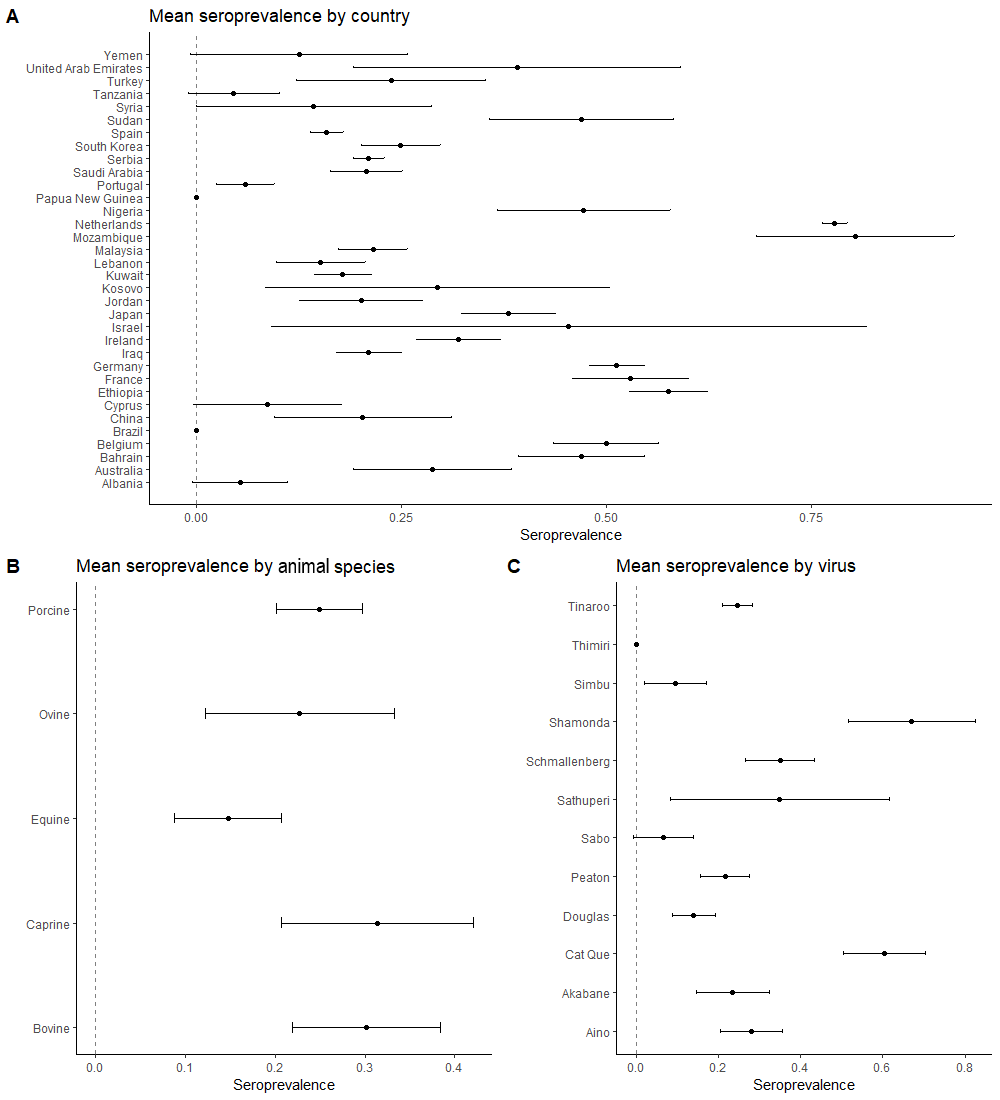

Supplement: Supplementary file 1 [file viruses-16-00294-s001.zip › Supplementary Figure S1. Mean seroprevalence.png]
